# A single-cell atlas of the myometrium in human parturition

**DOI:** 10.1172/jci.insight.153921

**Published:** 2022-03-08

**Authors:** Roger Pique-Regi, Roberto Romero, Valeria Garcia-Flores, Azam Peyvandipour, Adi L. Tarca, Errile Pusod, Jose Galaz, Derek Miller, Gaurav Bhatti, Robert Para, Tomi Kanninen, Ola Hadaya, Carmen Paredes, Kenichiro Motomura, Jeffrey R. Johnson, Eunjung Jung, Chaur-Dong Hsu, Stanley M. Berry, Nardhy Gomez-Lopez

**Affiliations:** 1 Perinatology Research Branch, Division of Obstetrics and Maternal-Fetal Medicine, Division of Intramural Research, Eunice Kennedy Shriver National Institute of Child Health and Human Development, National Institutes of Health, US Department of Health and Human Services (NICHD/NIH/DHHS), Bethesda, Maryland, and Detroit, Michigan, USA.; 2 Department of Obstetrics and Gynecology and; 3 Center for Molecular Medicine and Genetics, Wayne State University School of Medicine, Detroit, Michigan, USA.; 4 Department of Obstetrics and Gynecology, University of Michigan, Ann Arbor, Michigan, USA.; 5 Department of Epidemiology and Biostatistics, Michigan State University, East Lansing, Michigan, USA.; 6 Detroit Medical Center, Detroit, Michigan, USA.; 7 Department of Computer Science, Wayne State University College of Engineering, Detroit, Michigan, USA.; 8 Department of Physiology and; 9 Department of Biochemistry, Microbiology and Immunology, Wayne State University School of Medicine, Detroit, Michigan, USA.

**Keywords:** Cell Biology, Reproductive Biology, Bioinformatics, Obstetrics/gynecology

## Abstract

Parturition is a well-orchestrated process characterized by increased uterine contractility, cervical ripening, and activation of the chorioamniotic membranes; yet, the transition from a quiescent to a contractile myometrium heralds the onset of labor. However, the cellular underpinnings of human parturition in the uterine tissues are still poorly understood. Herein, we performed a comprehensive study of the human myometrium during spontaneous term labor using single-cell RNA sequencing (scRNA-Seq). First, we established a single-cell atlas of the human myometrium and unraveled the cell type–specific transcriptomic activity modulated during labor. Major cell types included distinct subsets of smooth muscle cells, monocytes/macrophages, stromal cells, and endothelial cells, all of which communicated and participated in immune (e.g., inflammation) and nonimmune (e.g., contraction) processes associated with labor. Furthermore, integrating scRNA-Seq and microarray data with deconvolution of bulk gene expression highlighted the contribution of smooth muscle cells to labor-associated contractility and inflammatory processes. Last, myometrium-derived single-cell signatures can be quantified in the maternal whole-blood transcriptome throughout pregnancy and are enriched in women in labor, providing a potential means of noninvasively monitoring pregnancy and its complications. Together, our findings provide insights into the contributions of specific myometrial cell types to the biological processes that take place during term parturition.

## Graphical abstract

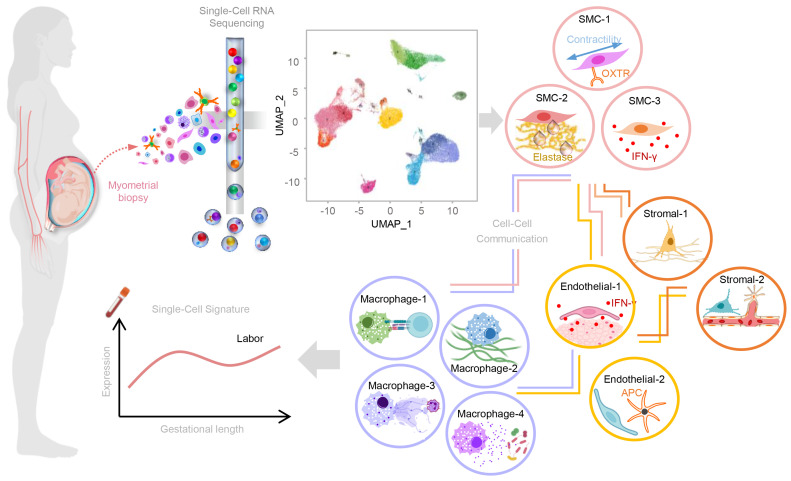

## Introduction

Labor is a multistage process including physiological, biochemical, endocrinological, and immunological pathways that occur in the mother and/or fetus, culminating in a successful delivery. This complex effort involves extrauterine and intrauterine components, wherein the latter consists of increased uterine contractility, cervical ripening (dilatation and effacement), and decidual/membrane activation (1–3). In most cases, labor is considered a state of physiological inflammation (4–8) as the majority of women undergoing spontaneous labor at term do not present with microbial invasion of the amniotic cavity (9, 10). This concept is supported by a growing body of evidence showing that an increase in cellular and soluble inflammatory mediators occurs in the uterine tissues (11–17), cervix (4, 12–15, 18, 19), decidua (7, 13, 14, 20–24), and chorioamniotic membranes (13, 14, 20–22) during labor. In addition to this local inflammation, labor is accompanied by a maternal systemic inflammatory response (15, 25–28). Yet, the process of labor is such that the utilization of high-dimensional approaches, i.e., transcriptomics, proteomics, metabolomics, and cytomics, is required to fully appreciate its complexity.

The uterus is a contractile organ essential to the common pathway of labor (1–3, 29). Anatomically, the uterus can be divided into regions that differ according to their location, composition, and functionality (30–40). The upper region, termed the corpus, comprises the largest proportion of smooth muscle cells that progressively decreases throughout the lower uterine segment up to the cervix (31, 40), which connects to the vagina. The lower uterine segment undergoes massive structural changes as gestation progresses due to increasing mechanical stress and cellular dynamics (30, 31, 41, 42). Moreover, this is the preferred site of hysterotomy during cesarean section (30, 43, 44) and is thus largely utilized to study the physiology of labor. Indeed, prior bulk transcriptomic investigations of the lower uterine segment have provided an overview of the myometrial molecular pathways implicated in this process. Initially, microarray data revealed that labor includes inflammatory responses such as cytokine and chemokine signaling, hypoxia, angiogenesis, and neutrophil signatures as well as expected pathways such as muscle function (45–49). Subsequent in silico analyses and meta-analyses confirmed this inflammatory profile and further sought to identify specific genes that could be key regulators of labor (49, 50). Particular interest has been given to known contraction-associated genes, with epigenetic surveys exploring the DNA methylation status of prostaglandins and their receptors (51). More recently, high-throughput RNA sequencing (RNA-Seq) has been applied to provide a better understanding of lower uterine segment gene expression by identifying a large number of transcripts that are associated with labor (52). A combined approach utilizing multiple available uterine transcriptomic data sets provided a thorough description of labor-associated pathways by confirming known inflammatory signatures as well as by identifying novel processes (53). The above studies provide a glimpse into the tissue-wide gene expression dynamics associated with the shift from quiescence to a contractile state (i.e., labor). However, the uterine tissues represent a heterogeneous landscape involving multiple cell types; thus, a more comprehensive investigation of cell type–specific transcriptional activity, which is not feasible with bulk RNA-Seq, is required to elucidate key biological pathways implicated in the molecular underpinnings of labor.

Single-cell RNA-Seq (scRNA-Seq) has emerged as a highly sensitive technique for determining the features of individual cells derived from whole tissues (54, 55). Previous investigations in humans and mice applied scRNA-Seq to dissect the cell type composition of the placenta and decidua in normal and complicated pregnancies (56–61). Indeed, we have utilized scRNA-Seq to decipher the cellular landscape of human parturition in the placental tissues, including the chorioamniotic membranes, as well as the contributions of each cell type to the common pathway of labor (62). Furthermore, we discovered that placental (28) and parturition-associated (62) single-cell signatures could be monitored in the peripheral blood, indicating that the local process of labor extends into the maternal circulation. Yet, a single-cell atlas and evidence of labor-specific transcriptomic activity in the uterine tissues, which include the myometrium (i.e., the pacemaker of parturition, ref. 63), have yet to be generated.

Herein, we performed scRNA-Seq to generate a comprehensive cellular landscape of the human lower uterine segment at term. Importantly, we identified labor-specific transcriptomic activity of individual myometrial nonimmune and immune cell types, shared gene dysregulation and pathways, and cell-cell communications. Furthermore, by comparing single-cell expression data to previously generated bulk transcriptomics, we demonstrated agreement between these analyses, with the former having the ability to identify cell type–specific changes associated with labor. Moreover, deconvolution of bulk expression data allowed for further appreciation of the role of smooth muscle cells (SMCs) in myometrial contractility and in inflammation associated with labor. Last, single-cell signatures derived from the myometrium were quantified in the maternal circulation from the first trimester to term pregnancy and, more importantly, were enriched in women undergoing spontaneous labor at term.

## Results

### A single-cell atlas of the human myometrium in term pregnancy.

**Figure 1:**
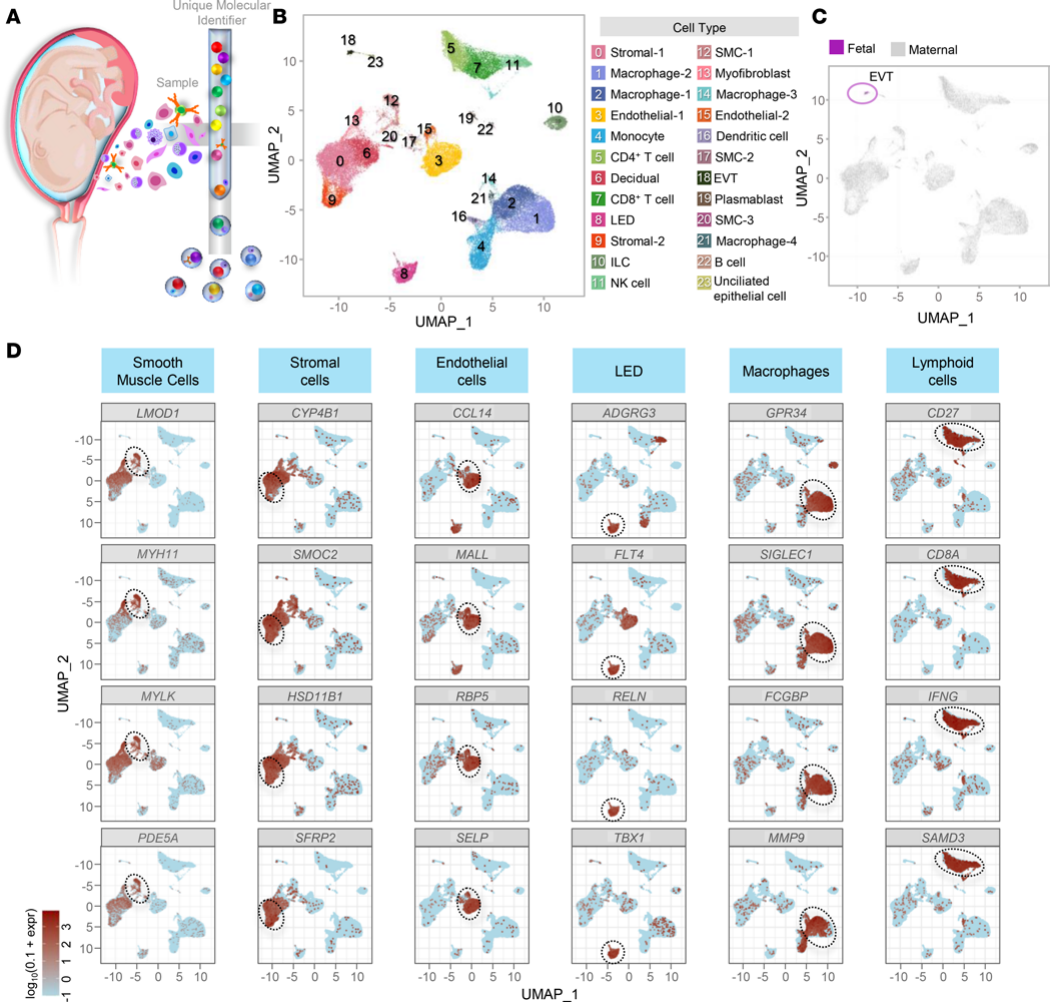
Single-cell transcriptional profile of the human myometrium. (**A**) Diagram illustrating the isolation of single cells from myometrial tissues at term (*n* = 24) and the formation of Gel Bead-in-Emulsion (GEM) as part of 10x Genomics scRNA-Seq. (**B**) Uniform manifold approximation and projection (UMAP) plot showing the cell types found in myometrial tissues. EVT, extravillous trophoblast; ILC, innate lymphoid cell; LED, lymphoid endothelial decidual cell; NK cell, natural killer cell; SMC, smooth muscle cell. (**C**) UMAP plot of the myometrial cell population color-coded to show maternal (gray) and fetal (violet) origin. (**D**) UMAP visualizations showing the expression of selected marker genes for major cell clusters, where expression is indicated by the colored bar. Major cell clusters are enclosed by a dotted line.

The common pathway of parturition is largely orchestrated by intrauterine cellular and molecular processes, most of which are still poorly understood. To fill this gap in knowledge, we generated a single-cell atlas of the lower uterine segment at term by performing scRNA-Seq in tissue samples collected from 24 laboring and nonlaboring women (Figure 1A and Supplemental Table 1; supplemental material available online with this article; https://doi.org/10.1172/jci.insight.153921DS1), which represents what we believe is the first and most comprehensive single-cell study of this maternal compartment. A total of 24 cell types that are displayed in the uniform manifold approximation and projection (UMAP) plot (Figure 1B and Supplemental Table 2) were identified by utilizing both computational and manual annotation methods, highlighting the heterogeneity of the myometrial tissues at term. The following maternal nonimmune cells were identified: SMCs (3 clusters), stromal cells (2 clusters), endothelial (3 clusters including LEDs; ref. 62), Decidual, Epithelial, and Myofibroblasts (Figure 1B). Moreover, maternal innate and adaptive immune cells were also present in the myometrial tissues, namely, macrophages (4 clusters), Monocytes, DCs, ILCs, NK cells, CD4^+^ and CD8^+^ T cells, and B cells/plasmablasts (Figure 1B). A small cluster of fetal cells (EVTs) was also found in the myometrial tissues (Figure 1C), which may represent placental cells attached to the endometrial lining. Representative transcripts utilized for cell type identification of the main clusters are shown in Figure 1D. Hence, we provide a single-cell atlas of the myometrium in term pregnancy, which was further utilized to interrogate the molecular underpinnings of parturition.

**Figure 2:**
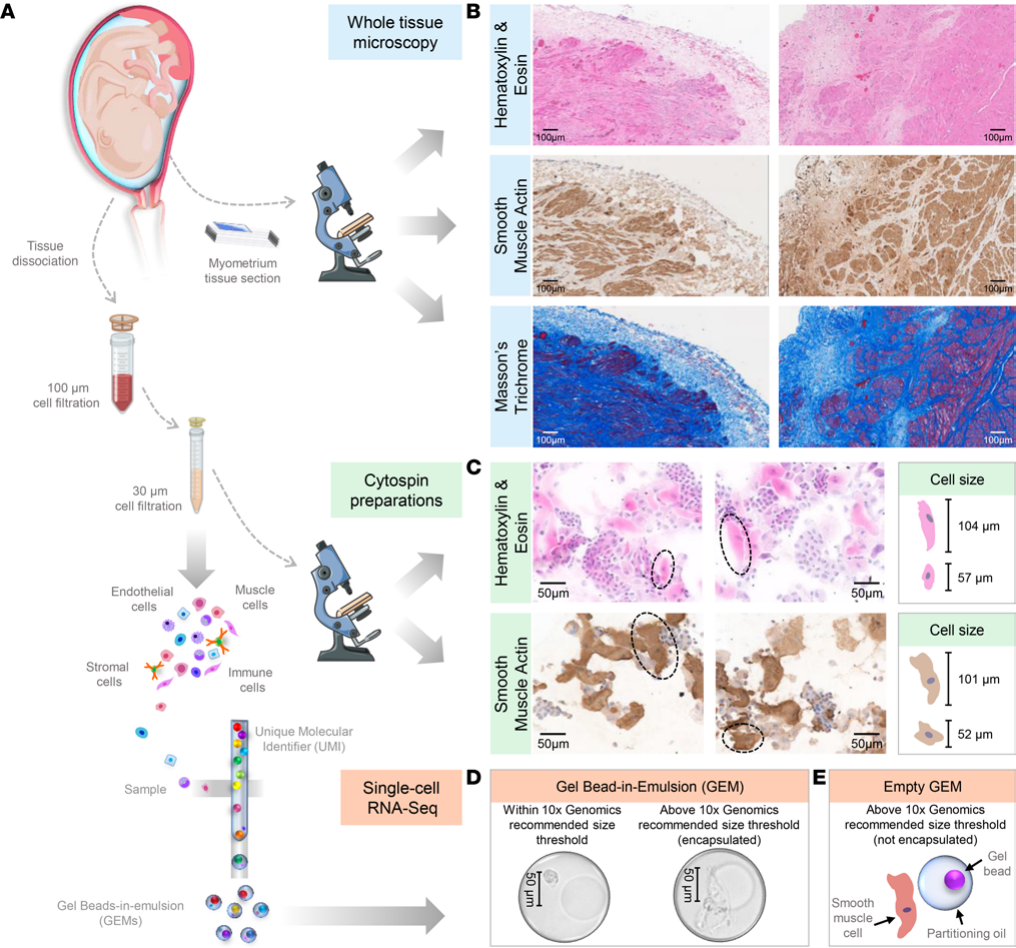
SMC population of the human myometrium. (**A**) Diagram illustrating the histological identification of the smooth muscle cell (SMC) population among myometrial cells before and after tissue dissociation (*n* = 24) for scRNA-Seq experiments. (**B**) Representative images from various sections of myometrial tissues showing (top) H&E staining, where the cell nuclei and cytoplasm are stained purple and pink, respectively; (middle) 3,3′-diaminobenzidine (DAB) immunohistochemistry staining for smooth muscle actin (brown); and (bottom) Masson’s trichrome staining showing collagen (blue) and muscle fibers (red). Scale bars at 100× original magnification: 100 μm. (**C**) Representative images of cells obtained after myometrial tissue dissociation stained using (top) H&E and (bottom) DAB immunohistochemistry staining for smooth muscle actin (brown). Scale bars at 200× original magnification: 50 μm. (**D**) Microscopic visualization of Gel Bead-in-Emulsion (GEM) successfully encapsulating single cells with SMC-like morphology that are within (left) and above (right) the 10x Genomics recommended size threshold. Scale bars at 200× original magnification: 50 μm. (**E**) Diagram illustrating a SMC above the 10x Genomics recommended size threshold that cannot be successfully encapsulated within the GEM.

**Figure 3:**
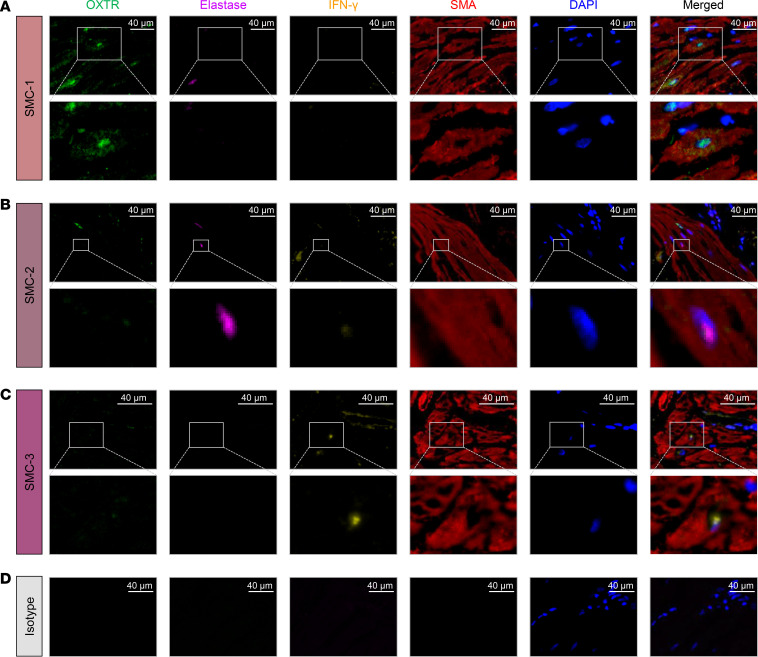
Spatial identification of SMC subpopulations. (**A**–**C**) Representative images showing the protein expression of OXTR (oxytocin receptor, green), elastase (purple), IFN-γ (orange), and smooth muscle actin (SMA, red) in myometrial tissues from women who delivered at term in labor (*n* = 2) to identify (**A**) SMC-1, (**B**) SMC-2, and (**C**) SMC-3 subpopulations. Nuclear staining is shown in blue (4′,6-diamidino-2-phenylindole, DAPI). Merged images showing the coexpression of different markers are also included. Images were scanned at 40× original magnification (scale bar = 40 μm; top panel) with digital zoom (**A** and **C**, original magnification, 3×–5×; and **B**, original magnification, 10×) (bottom panel). (**D**) Representative images of a slide stained with isotype (negative control). Images were scanned at 40× original magnification (scale bar = 40 μm).

The basic functional unit of the myometrium is the SMC, whose size can vary depending on anatomical location and activation status. Therefore, we next explored whether the utilized single-cell technology allowed for the capture of SMCs of a wide range of sizes, given that the theoretical size limit of single-cell encapsulation is 50–60 μm, as determined by the width of the microfluidic channel (64). First, histological evaluation of the myometrial biopsies was performed before and after single-cell preparation (Figure 2, A–C). As expected, smooth muscle tissue was visualized in all 24 biopsies by conventional (H&E) and special (smooth muscle actin and Masson’s trichrome) staining (Figure 2B). Yet, other cell types were also observed, highlighting the heterogeneous cellular composition of this tissue (Figure 2B). After tissue dissociation, SMCs were also evident in single-cell suspensions; however, their sizes varied (50–120 μm) (Figure 2C). Both SMCs falling below the single-cell size threshold and those above this theoretical limit could be encapsulated, as shown in Figure 2D. Yet, a proportion of these cells falling above the single-cell size threshold could not be captured (Figure 2E). Therefore, cell size represents a limitation of single-cell microfluidic techniques, which provides an explanation for the reduced frequency of SMCs in myometrial single-cell suspensions. Regardless, the presence of SMC clusters in our single-cell data set allowed us to characterize them into 3 subsets (Figure 1B). Further Gene Ontology (GO) analysis revealed that different biological processes were enriched in each of these subsets: SMC-1, smooth muscle contraction; SMC-2, neutrophil biology; and SMC-3, response to IFN-γ (Supplemental Figure 1, A–C, and Supplemental Table 3). Multiplex immunofluorescence confirmed that some SMCs expressed oxytocin receptor (OXTR), a classical contractility-associated protein (65), but lacked the expression of elastase (a stereotypical product of activated neutrophils; ref. 66) or IFN-γ (Figure 3A). Furthermore, other SMCs expressed either elastase or IFN-γ but not OXTR (Figure 3, B and C). Isotype controls showed no unspecific signal (Figure 3D). It is worth mentioning that only a subset of SMCs was accounted for by the 3 distinct phenotypes identified herein, suggesting that additional characterization of such cells is required. Further analysis also showed that the different subsets of stromal and macrophage cell types may display different functions in the human myometrium (Supplemental Figures 2 and 3 and Supplemental Tables 4 and 5). It is important to clarify that the macrophage clusters described in our study do not correspond to the M1-M2 macrophage polarization phenotypes described at the materno-fetal interface of women with preterm labor (67, 68). In the current study, macrophage clusters were involved in diverse immune processes: Macrophage-1, antigen presentation; Macrophage-2, wound healing; Macrophage-3, neutrophil response; and Macrophage-4, response to bacterium (Supplemental Figure 3). Therefore, scRNA-Seq allowed for the identification of multiple cell types with distinct functionality in the human myometrium.

### Labor-associated single-cell transcriptional activity and cell-cell communications in the myometrium.

**Figure 4:**
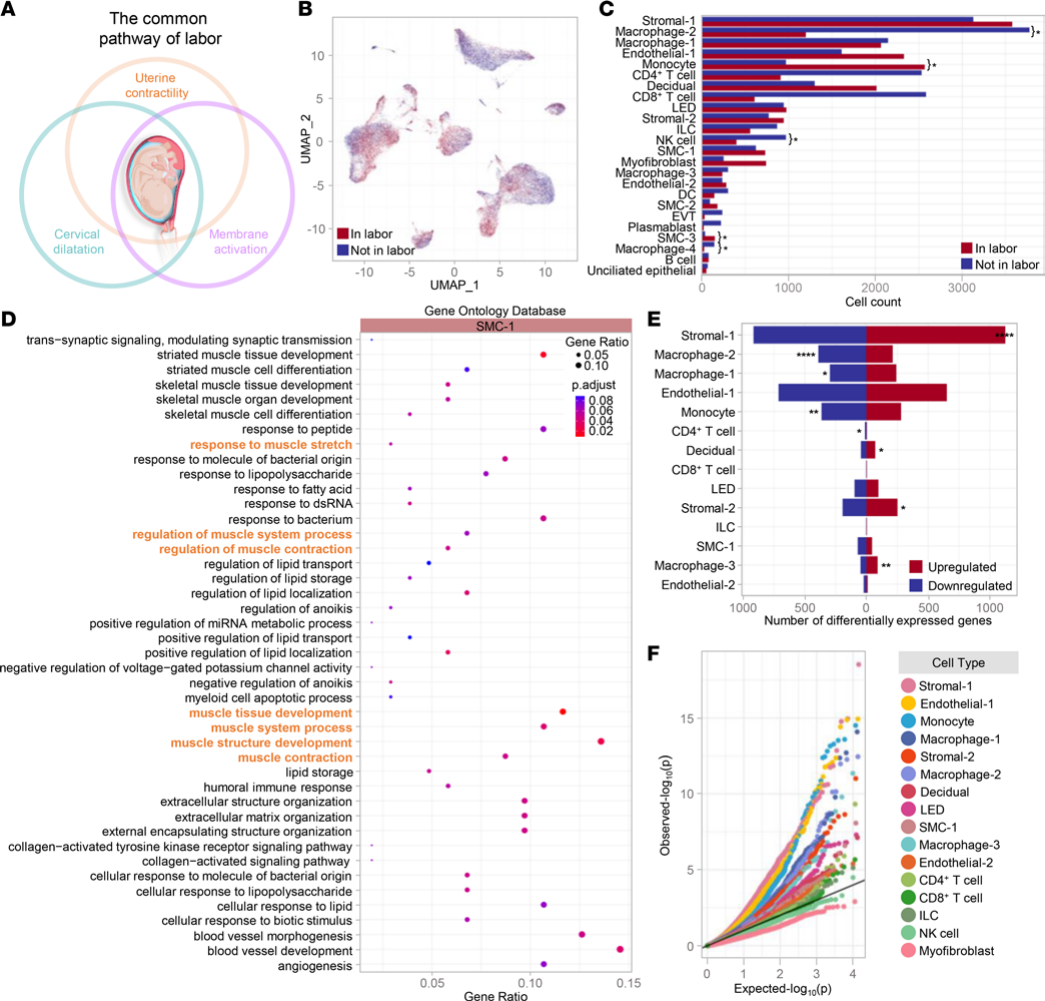
The myometrial transcriptomic profile in labor. (**A**) Diagram illustrating the common pathway of labor. (**B**) Uniform manifold approximation and projection (UMAP) plot showing cell clusters in the myometrial tissues from women in labor (*n* = 11, red) and not in labor (*n* = 13, blue). (**C**) Bar plots showing the number of cells for each cell type in the myometrial tissues from women in labor (red) and not in labor (blue). The comparison of the numbers of each cell type between the 2 study groups was performed using a 2-sided binomial test where *q* < 0.05 is considered significant, as represented by an asterisk (*). (**D**) ClusterProfiler dot plot showing the Gene Ontology (GO) biological processes that are enriched in labor based on differentially expressed genes (DEGs) in the SMC-1 cell type (*q* < 0.1), where the size and color of the dots represent enrichment score and significance level, respectively. The overrepresentation analysis was performed based on the 1-sided Fisher’s exact test (ClusterProfiler). GO terms with *q* < 0.1 were selected. Highlighted in orange are pathways associated with uterine contractility. (**E**) Bar plot showing the number of labor-associated DEGs for each cell type, where red and blue indicate upregulated and downregulated DEGs, respectively. The comparison between upregulated and downregulated DEGs was calculated using a 2-sided binomial test, where *q* < 0.05 is considered significant. *q < 0.05, **q < 0.01, ****q < 0.0001. (**F**) Quantile-quantile plot showing differential expression of genes analyzed for selected enriched cell types from the myometrial tissues. Deviation above the 1:1 line (solid black line) indicates enrichment. DC, dendritic cell; EVT, extravillous trophoblast; ILC, innate lymphoid cell; LED, lymphoid endothelial decidual cell; NK cell, natural killer cell; SMC, smooth muscle cell.

Uterine contractility is 1 of the 3 components of the common pathway of labor (Figure 4A). Therefore, we next assessed the effect of labor on the transcriptional activity of the uterine tissues at single-cell resolution. The overall effect of labor on each myometrial cell type is depicted in Figure 4B; all cell types were represented in tissues collected from both laboring and nonlaboring women. Yet, significant differences in cell counts were mainly observed in the innate immune cell compartment (macrophage clusters, Monocytes, and NK cells) as well as in SMC-3 when comparing labor and nonlabor tissues (Figure 4C). Differentially expressed genes (DEGs) in SMC-1 (the most abundant SMC cluster) are displayed in Supplemental Figure 4. GO analysis of these transcripts revealed enrichment of muscle contraction processes (Figure 4D), which is consistent with the involuntary contractile nature of the uterus. Further analysis revealed a large number of DEGs in both immune and nonimmune cells (Figure 4E and Supplemental Table 6), of which the top 10 are shown in Supplemental Figure 5. Labor transcriptional activity was upregulated in Stromal-1, Stromal-2, Macrophage-3, and Decidual cell types (Figure 4E), while such activity was downregulated in Macrophage-2, Monocyte, and Macrophage-1 cell types as well as in CD4^+^ T cells (Figure 4E). The most labor-impacted cell types were Stromal-1, Endothelial-1, Monocyte, Macrophage-2, Macrophage-1, Stromal-2, Macrophage-3, LED, SMC-1, Decidual, Endothelial-2, CD4^+^ T cells, CD8^+^ T cells, and ILCs (Figure 4F and Supplemental Table 6). The top 10 DEGs in response to labor in each cell type are shown in Supplemental Figure 5, suggesting distinct labor-specific modulation of transcriptional activity across cell types.

**Figure 5:**
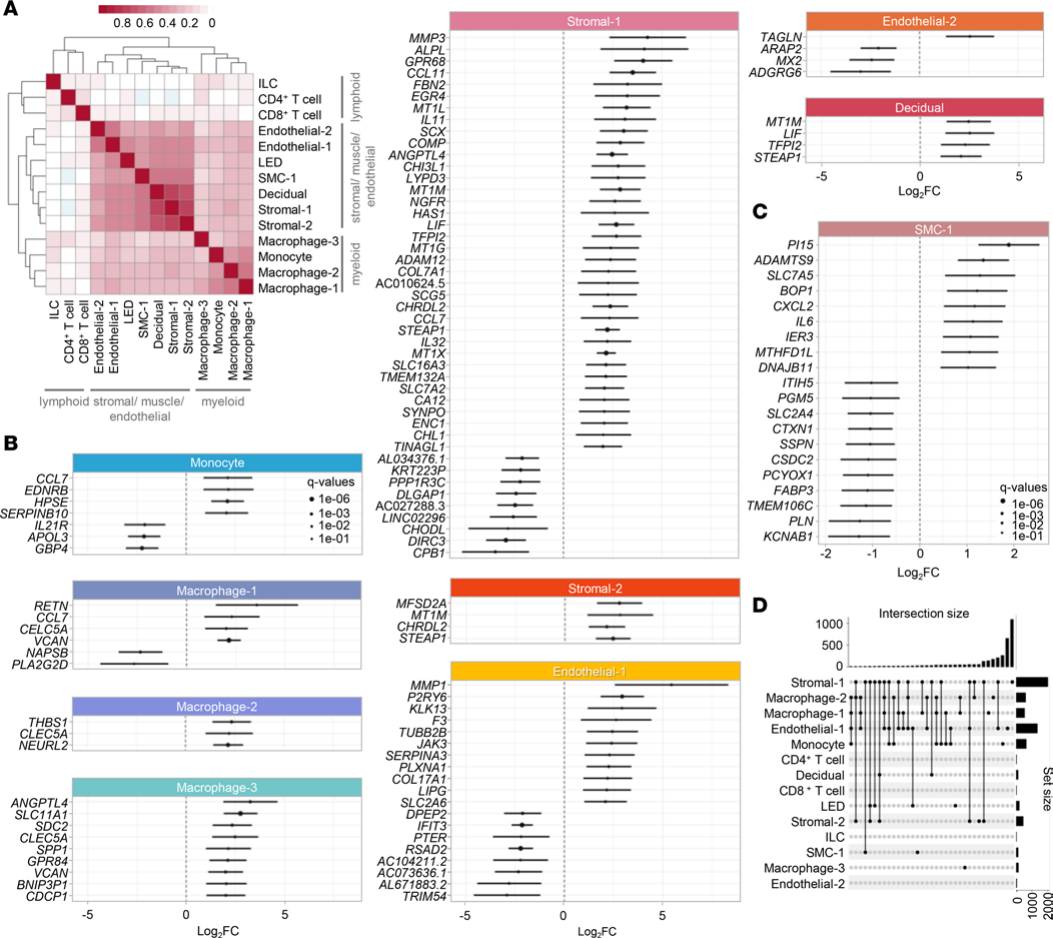
The effect of labor on the transcriptional activity of myometrial cell types. (**A**) Heatmap showing the log_2_(fold change) correlation among cell types from myometrial tissues (*n* = 24), where red and white blocks signify increased and decreased correlation, respectively. A 2-sided Spearman’s correlation test was used. (**B**) Forest plots showing upregulated and downregulated labor-associated differentially expressed genes (DEGs) in Monocyte, Macrophage-1, Macrophage-2, Macrophage-3, Stromal-1, Stromal-2, Endothelial-1, Endothelial-2, and Decidual cell types. DEGs shown are significant with FDR < 0.05 and absolute log_2_(fold change) ≥ 2. (**C**) Forest plot showing upregulated and downregulated labor-associated DEGs in SMC-1, which are significant with FDR < 0.05 and absolute log_2_(Fold change) ≥ 1. DEGs were identified based on the 2-sided Wald’s test on the negative binomial distribution (DESeq2). (**D**) UpSet plot showing intersections between selected cell types based on common labor-associated DEGs, where connected black dots represent overlapping cell types and vertical bars in intersection set show degree of overlap. DEGs were selected based on *q* < 0.1. ILC, innate lymphoid cell; LED, lymphoid endothelial decidual cell; SMC, smooth muscle cell.

**Figure 6:**
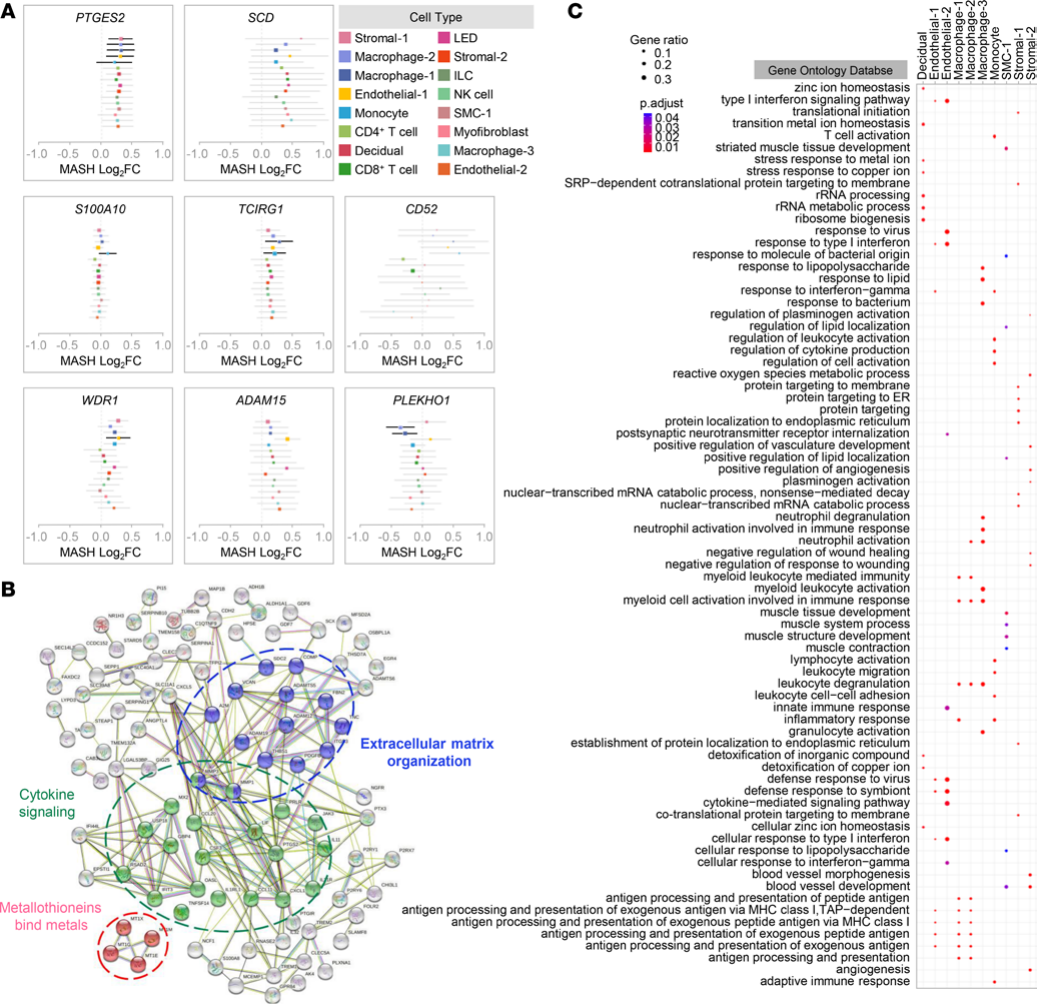
Genes and pathways involved in myometrial physiology. (**A**) Meta plots showing differential expression of representative genes (defined as posterior means and variance of effects) across selected cell types obtained using MASH analysis. Bold lines are used to highlight the specific contributions of selected cell types. (**B**) STRING analysis plot showing interactions of labor-associated differentially expressed genes (DEGs) in the myometrial tissues (*n* = 24) derived from the Reactome database, where subnetworks in blue represent extracellular matrix organization, green represent cytokine signaling, and pink represent response to metallothioneins bind metals. (**C**) ClusterProfiler dot plot showing biological processes enriched for labor-associated DEGs in the Decidual, Endothelial-1, Endothelial-2, Macrophage-1, Macrophage-2, Macrophage-3, Monocyte, SMC-1, Stromal-1, and Stromal-2 cell types of myometrial tissues based on the overrepresentation analysis, where the size and color of the dots represent enrichment score and significance level, respectively. A 1-sided Fisher’s exact test (ClusterProfiler) was used. Enriched GO terms with *q* < 0.05 were selected. ILC, innate lymphoid cell; LED, lymphoid endothelial decidual cell; NK cell, natural killer cell; SMC, smooth muscle cell.

We next sought to more closely investigate the shared and nonshared effects of labor across selected cell types. First, a correlation analysis across the labor-associated genes for each pair of cell types revealed that the differential gene expression was clustered into 3 major cell lineage groups: myeloid, stromal/muscle/endothelial, and lymphoid (Figure 5A). In myeloid cells, the effect of labor was more similar between Macrophage-1 and Macrophage-2, while Macrophage-3 displayed a distinct pattern. By contrast, the effect of labor in lymphoid cells was distinct from that observed in myeloid cells; therefore, there was a poor correlation between lymphoid and myeloid cells. The stromal/muscle/endothelial cell types displayed the greatest shared effects of labor represented in the larger cluster in Figure 5A. Forest plots were generated to show the individual genes upregulated and downregulated by labor in selected cell types (Figure 5, B and C), which together with the data displayed in the UpSet plot (Figure 5D) highlight those transcripts that are labor specific for each cell type. Yet, there was substantial labor-shared gene dysregulation among cell types, as indicated by fold change correlation analysis as illustrated in Figure 5A as well as by multivariate adaptive shrinkage (MASH) analysis as shown in Supplemental Figure 6. Such analysis was also used to highlight cell type–specific labor-associated gene dysregulation (Figure 6A). Search Tool for the Retrieval of Interacting Genes/Proteins (STRING) pathway analysis of the top DEGs revealed enrichment of pathways from the Reactome database, which included cytokine signaling, extracellular matrix organization, and metallothioneins bind metals (Figure 6B and Supplemental Figure 7A). Consistently, pathway analyses performed by using the Kyoto Encyclopedia of Genes and Genomes (KEGG) and GO databases depicted similar innate immunity-driven processes (Supplemental Figure 7, B and C). A network representation of the protein-protein interactions (STRING database) where the nodes are either DEGs shared among cell types or genes with a high degree of centrality is shown in Supplemental Figure 8.

To further understand the contribution of each myometrial cell type to the above biological processes, pathway analysis was performed in DEGs from the top 10–11 cell types most affected by labor. GO, KEGG, and Reactome pathway analyses revealed that different cell types participate in diverse processes (Figure 6C and Supplemental Figures 9 and 10). For example, Macrophage-1 is involved in antigen presentation whereas Macrophage-3 is implicated in neutrophil response (Figure 6C and Supplemental Figure 10). Furthermore, whereas Endothelial-1 participates in antigen presentation processes, Endothelial-2 is involved in IFN-mediated responses (Figure 6C and Supplemental Figures 9 and 10).

**Figure 7:**
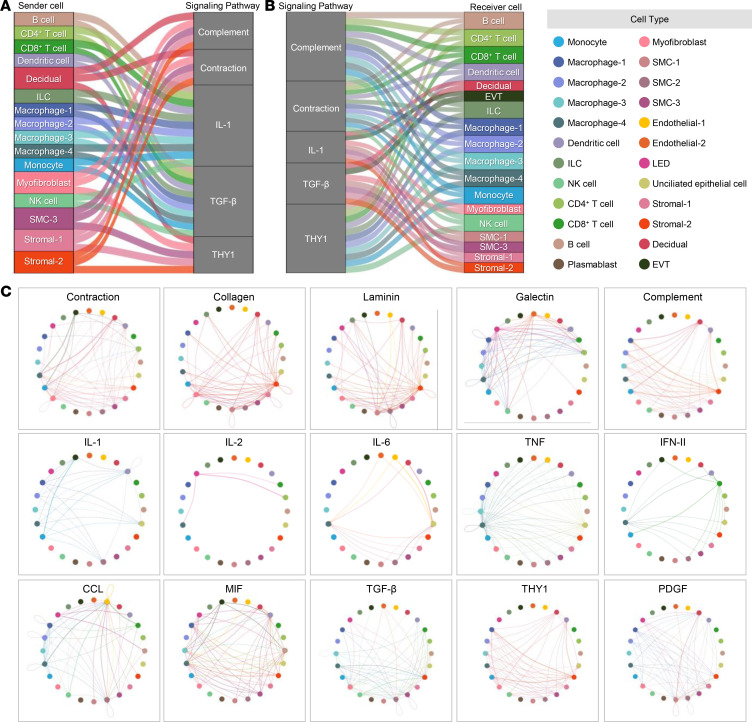
Intercellular communication in the human myometrium. (**A** and **B**) Alluvial plots showing (**A**) major outgoing signals from sender cells to signaling pathways and (**B**) incoming signals to receiver cells in signaling pathways (contribution score > 0.7). The interactions show the contribution of cells in sending and receiving signals. Sender and receiver cells are color-coded based on the myometrial cell types. (**C**) Circle plots showing the significant (*P* < 0.05) cell-to-cell communications with probability > 0.9 for each labor-associated signaling pathway. Each node represents a myometrial cell type, and connecting lines are color-coded based on the sender cell. EVT, extravillous trophoblast; ILC, innate lymphoid cell; LED, lymphoid endothelial decidual cell; NK cell, natural killer cell; SMC, smooth muscle cell.

Next, we further leveraged our data to explore cell-cell communications during the process of labor. Alluvial plots show the major communication processes and cell types that serve as senders or receivers (Figure 7, A and B, and Supplemental Table 7). Both immune and nonimmune cell types can serve as senders and/or receivers of labor-associated signaling. Interestingly, specific cell types, i.e., Decidual, Myofibroblast, SMC-3, Stromal-1, and Stromal-2 served as senders of signals for the process of contraction, extracellular matrix (e.g., collagen and laminin), and complement, while immune cell types such as B cells, T cells, ILCs, macrophages, Monocytes, and NK cells served as senders for IL-1 signaling (Figure 7A). By contrast, signals related to the process of contraction were received by immune cells, namely, B cells, T cells, DCs, macrophage cell types, Monocytes, and NK cells (Figure 7B). Yet, Decidual, EVT, and Myofibroblasts, as well as SMCs and stromal cell types, acted as receivers for IL-1 signaling (Figure 7B). Circle network plots display more detailed cell-cell communications for each signaling pathway including all significant cell types explored. Some pathways, such as contraction, collagen, and laminin, were shown to be strongly represented in our labor data set (Figure 7C).

Collectively, these data show that the process of labor profoundly impacts the transcriptome of nonimmune and immune cell types in the human myometrial tissues and that several signaling pathways are governed by distinct cell types. Such pathways include both immune and nonimmune processes such as contraction and innate immune activation, the latter primarily driven by infiltrating immune cells. These findings not only support the concept that immune cell–derived inflammation is a central component of human labor but also provide evidence that myometrial nonimmune cells participate in such a process.

### scRNA-Seq and deconvolution analysis provide deeper insight into labor dynamics than bulk transcriptomics.

**Figure 8:**
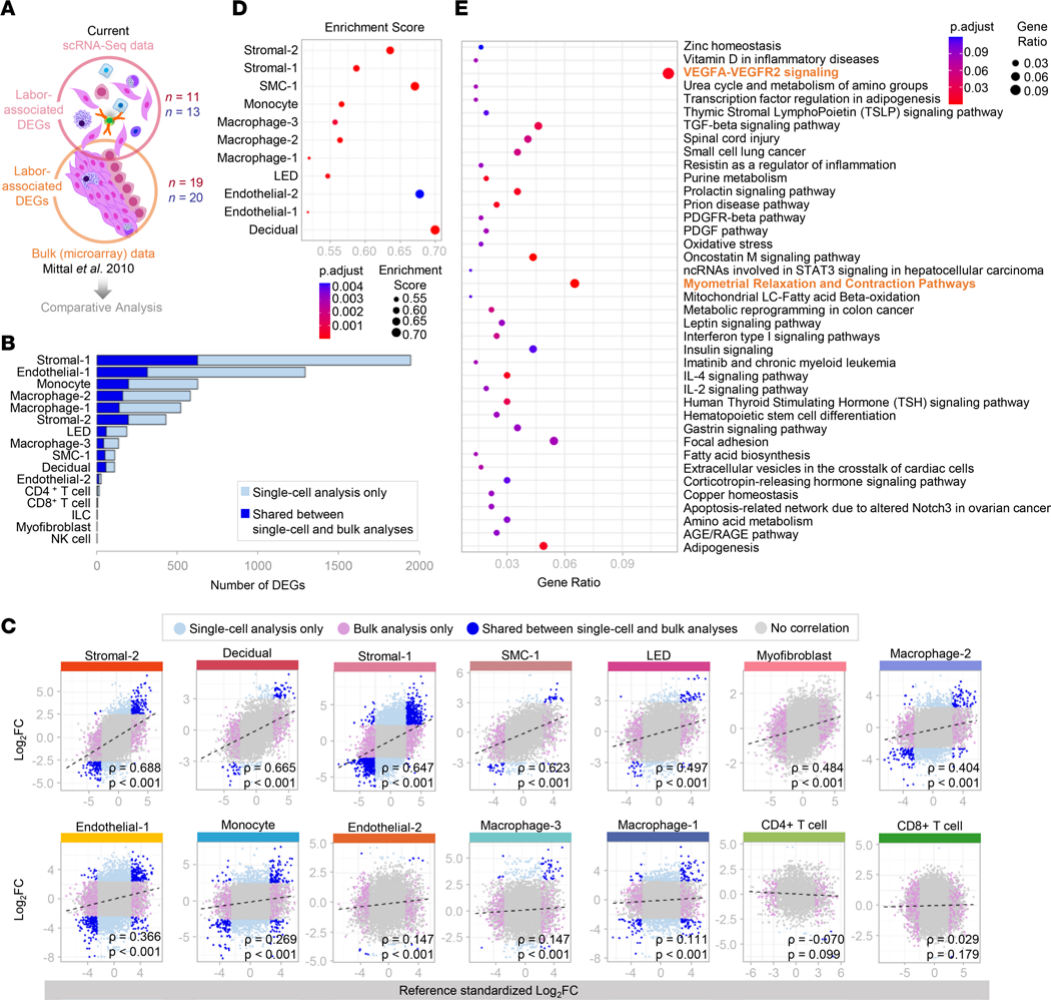
Comparative transcriptomic analysis of myometrial single-cell and bulk data at term in labor. (**A**) Comparative analysis of labor-associated differentially expressed genes (DEGs) from myometrial tissues using single-cell (*n* = 11 in labor, red; *n* = 13 not in labor, blue) and bulk microarray analysis (*n* = 19 in labor, red; *n* = 20 not in labor, blue) as previously reported (Mittal et al. 2010) (46). (**B**) Bar plot showing numbers of labor-associated DEGs detected from single-cell analysis alone (light blue) compared to DEGs shared between single-cell and bulk analyses (dark blue). A 2-sided Wald’s test was performed based on negative binomial distribution. (**C**) Scatter plots showing the log_2_(Fold change) associated with labor in bulk microarray (*x* axis) and single-cell (*y* axis) analyses by cell type. DEGs shown were obtained using only single-cell analysis (light blue) or bulk analysis (lavender), shared by both single-cell and bulk analyses (dark blue), or not differentially expressed between the 2 groups (gray). Correlations between data sets were determined using 2-sided Spearman’s correlation test. Black dashes represent the regression line. (**D**) ClusterProfiler dot plot showing cell types enriched in labor-associated DEGs from bulk analysis, where dot size and color represent enrichment score and significance level, respectively. Significant cell types (*q* < 0.1) were identified based on GSEA (1-sided Kolmogorov-Smirnov test). (**E**) ClusterProfiler dot plot showing biological pathways from Wikipathways enriched for labor-associated DEGs combining smooth muscle cell-1 (SMC-1) single-cell analysis and deconvolution analysis of bulk gene expression data of laboring myometrial tissues. Significant pathways were identified based on overrepresentation analysis using the 1-sided Fisher’s exact test. Pathways with *q* < 0.1 were selected. The top 2 pathways most enriched during labor are in orange. ILC, innate lymphoid cell; LED, lymphoid endothelial decidual cell; NK cell, natural killer cell.

To further unravel the cell type–specific contributions to the process of labor, we performed a fold change–based comparative analysis between our scRNA-Seq data and previously generated bulk microarray data (46) of the uterine tissues (Figure 8A). scRNA-Seq analysis identified a substantially larger number of labor- and cell type–specific DEGs than the number inferred in microarray data (Figure 8B). The most affected cell types were Stromal-1, Endothelial-1, Monocyte, Macrophage-2, Macrophage-1, and Stromal-2 (Figure 8B). When considering only those labor-associated DEGs shared between data sets, significant positive correlations were observed among nonimmune and immune cells such as Stromal-2, Decidual, Stromal-1, SMC-1, LED, Myofibroblast, Macrophage-2, Endothelial-1, Monocyte, Endothelial-2, Macrophage-3, and Macrophage-1 (Figure 8C and Supplemental Figure 11). A nonsignificant negative correlation was observed for CD4^+^ T cells (Figure 8C and Supplemental Figure 11). Gene set enrichment analysis (GSEA) of labor-associated DEGs is displayed in Figure 8D. Notably, Stromal-2, SMC-1, Decidual, and Endothelial-2 cell types showed the greatest enrichment scores (Figure 8D). Therefore, this comparative analysis underscores the enhanced capability of single-cell technology to reveal hitherto unappreciated labor-specific transcriptomic activity from different cell types found in the myometrium.

To further appreciate the role of uterine SMCs in the process of labor, we performed deconvolution analysis by intersecting our SMC single-cell signature with previously generated bulk microarray data (46) (Supplemental Figure 12A) as a means of overcoming the underrepresentation of SMCs in our scRNA-Seq data. Pathway analysis of the SMC-1 cell type combined with those genes inferred from the deconvolution analysis revealed enrichment for myometrial relaxation and contraction pathways as well as VEGFA-VEGFR2 signaling in labor (Figure 8E). Specific DEGs implicated in the myometrial relaxation and contraction pathways are displayed in Supplemental Figure 12B. The contribution of inferred “smooth muscle cell-1” to labor-specific gene expression compared to the other cell types is displayed in Supplemental Figure 13A. Although the primary function of such inferred cell type is related to SMC contraction as shown by STRING analysis (Supplemental Figure 13B), these cells can also express inflammatory mediators such as *IL1B* and *IL6*, as well as other mediators (Supplemental Figure 12B and Supplemental Figure 13A). Thus, deconvolution analysis confirms the contractile nature of uterine SMCs and extends the participation of these cells to the inflammatory process taking place in parturition.

### Uterine single-cell signatures are modulated in the maternal circulation throughout pregnancy and with labor.

**Figure 9:**
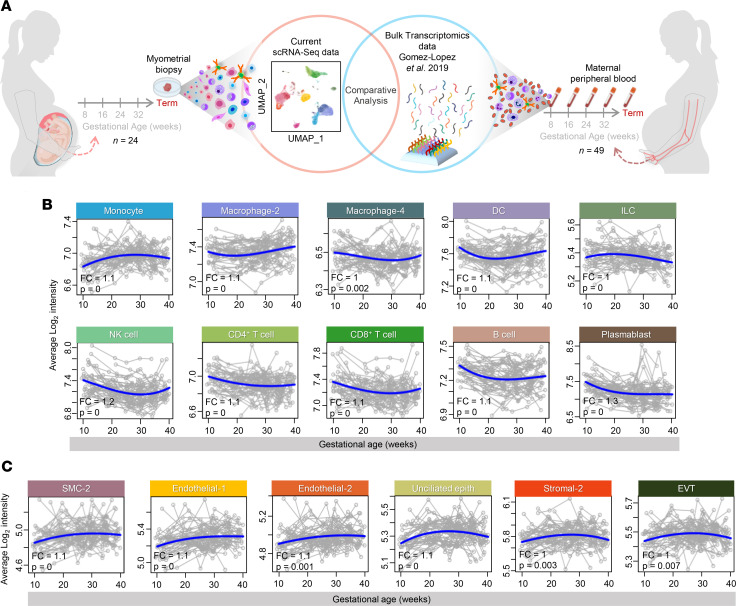
Comparative analysis of single-cell signatures from myometrial tissues and bulk transcriptomics from maternal peripheral blood throughout gestation. (**A**) Experimental design showing comparative analysis of genes from scRNA-Seq of myometrial tissues of women who delivered at term (*n* = 24) versus bulk transcriptomics of maternal peripheral blood collected throughout gestation in the absence of labor (*n* = 49) from a previously reported data set (Gomez-Lopez et al. 2019) (69). (**B** and **C**) Signature analysis plots showing changes in the average expression of labor-associated differentially expressed genes (DEGs) (blue line) related to (**B**) immune cells and (**C**) nonimmune cells from maternal peripheral blood collected throughout gestation compared to myometrial tissues collected at term. Each gray line corresponds to 1 sample. *P* value and FC are reported from the longitudinal analysis. DC, dendritic cell; EVT, extravillous trophoblast; ILC, innate lymphoid cell; NK cell, natural killer cell; SMC, smooth muscle cell; FC, fold change.

To generate a potential noninvasive approach to monitor pregnancy and its complications, we next intersected our single-cell signatures derived from the myometrial tissues with available cellular transcriptomic data from the maternal circulation of mothers throughout gestation who delivered at term without labor from Gomez-Lopez et al. 2019 (69) (Figure 9A). Such a strategy has been previously utilized by us and others, using placenta-derived single-cell signatures (28, 62, 70–90). Multiple single-cell signatures from immune and nonimmune uterine cell types were modulated in the maternal circulation throughout gestation (Figure 9, B and C, and Supplemental Table 8). Consistent with the concept of an immunological clock during pregnancy (27, 91–93), both innate and adaptive immune cell signatures such as Monocyte, Macrophage-2, Macrophage-4, DC, ILC, NK cell, CD4^+^ and CD8^+^ T cell, B cell, and Plasmablast were significantly modulated during gestation (Figure 9B). Interestingly, signatures of nonimmune cell types were also significantly modulated in the maternal circulation, namely SMC-2, Endothelial-1, Endothelial-2, Unciliated Epithelial, Stromal-2, and EVT (Figure 9C).

**Figure 10:**
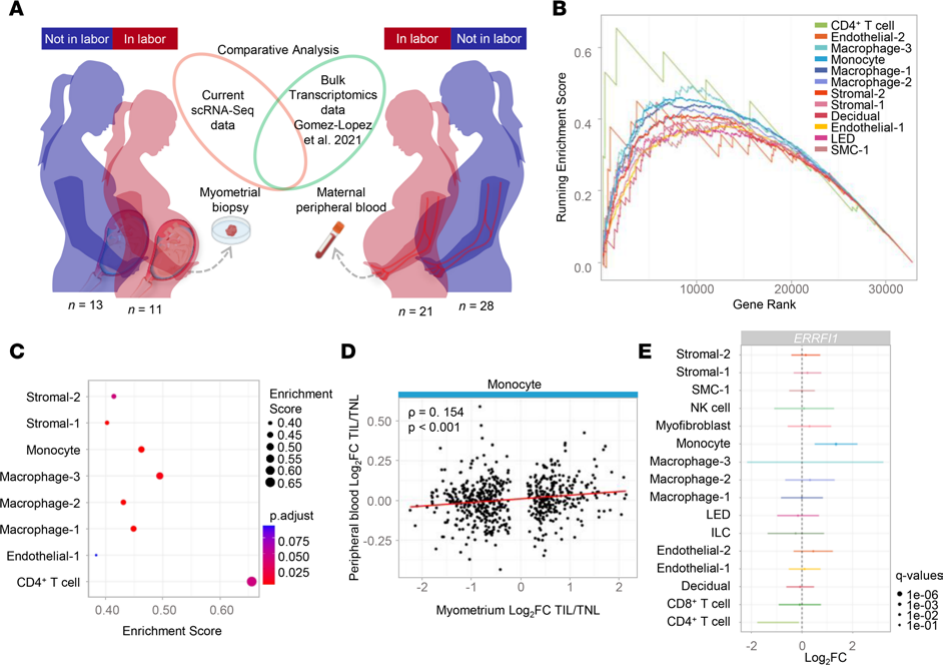
Comparative analysis of the single-cell transcriptomic profile of myometrial tissues and the bulk transcriptomic profile of maternal peripheral blood at term in labor versus term not in labor. (**A**) Diagram illustrating the comparative analysis of myometrial tissues collected from women at term in labor (*n* = 11) and not in labor (*n* = 13) using scRNA-Seq compared with maternal peripheral blood collected from women at term in labor (*n* = 21) and not in labor (*n* = 28) using bulk transcriptomics from a previously reported data set (Gomez-Lopez et al. 2021) (94). (**B**) GSEA plot showing enrichment scores using the ranked list of differentially expressed genes (DEGs) comparing women at term in labor and not in labor from maternal peripheral blood and cell type annotations of labor-associated DEGs analyzed from myometrial tissues. A 1-sided Kolmogorov-Smirnov test was used. (**C**) ClusterProfiler dot plot showing gene enrichment in selected cell types, where the size and color of the dots represent enrichment score and significance level, respectively. A 1-sided Kolmogorov-Smirnov test was used. (**D**) Scatter plot showing labor fold changes detected as differentially expressed in myometrial monocytes (*x* axis) that were also detected in peripheral blood (*y* axis). A 2-sided Spearman’s correlation test was performed. (**E**) Forest plot showing cell type–specific association to the labor process for *ERRFI1*. Each dot represents the log_2_(fold change) when comparing term in labor and term not in labor groups, and the bars represent the 95% confidence interval. ILC, innate lymphoid cell; LED, lymphoid endothelial decidual cell; NK cell, natural killer cell; SMC, smooth muscle cell.

To further enhance the biological relevance of monitoring myometrium-derived single-cell signatures in the maternal circulation, we next evaluated labor-specific transcriptomic changes that are shared between the local and peripheral compartments. Whole-blood gene expression dysregulation derived at the time of labor from a separate patient cohort (*n* = 49) was obtained from Gomez-Lopez et al. 2021 (94) and was intersected with our single-cell signatures derived from the uterine tissues (Figure 10A). The GSEA of labor-associated myometrial signatures revealed enrichment of both immune and nonimmune cell types, including CD4^+^ T cell, Macrophage-3, Monocyte, Macrophage-1, Macrophage-2, Stromal-2, and Stromal-1, in the peripheral blood data set (Figure 10, B and C). In addition, a significant correlation for the Monocyte cell type was observed between labor-specific signatures in the myometrial tissues and maternal circulation (Figure 10D). Furthermore, several labor-specific DEGs were identified in immune and nonimmune cells, with the gene ERBB receptor feedback inhibitor 1 (*ERRFI1*) being specifically differentially expressed in monocytes in the maternal circulation (Figure 10E).

Collectively, these data indicate that single-cell signatures derived from the myometrial tissues can be utilized for the temporal tracking of maternal systemic cellular dynamics throughout gestation, can identify cell type–specific transcripts that are enriched during the physiological process of labor, and may potentially serve as biomarkers.

## Discussion

The cellular composition of the uterine layers (perimetrium, myometrium, and endometrium) varies throughout pregnancy as evidenced by histological studies (30, 31, 40, 95, 96). Indeed, the process of labor induces specific modifications, including the well-characterized infiltration of immune cells into the myometrium (7, 11–15, 97), uterine vascular remodeling (98), and changes in connective tissue (99), among others. These histological changes are in line with our current scRNA-Seq findings that demonstrate the complex and heterogeneous nature of the cellular compartment in the myometrial tissues and its modifications during the process of labor. Next, we further discuss the putative roles of the main scRNA-Seq–derived myometrial cell types in the physiological process of term parturition.

SMCs represent the functional unit of the myometrium, whose main role is to orchestrate the contractility of the uterine apparatus, one of the components of the common pathway of parturition (1–3, 29, 100). Consistent with such a role, in the current study, we showed that the transcriptomic activity of uterine SMCs was enriched for processes related to myometrial contraction and relaxation. Myometrial contraction is a complex process regulated by contraction-associated proteins (CAPs), such as OXTR, prostaglandin F2α receptor, prostaglandin E_2_ (PGE_2_) receptor, the primary gap junction protein connexin-43, and prostaglandin-endoperoxide H synthase 2 (PGHS-2, also known as cyclooxygenase-2 or COX-2), among others (65, 101–104). Contradictory results have been reported regarding the CAPs’ expression profiles between the upper and lower uterine segments (33, 37, 105–108); therefore, it is challenging to speculate whether such changes result in differential contractility rate between these 2 compartments (32, 109, 110). It is tempting to suggest that single-cell transcriptomics may allow for the discovery of specific processes taking place in the upper or lower uterine compartments. However, paired samples collected from the same patient would be required, jeopardizing the feasibility of such a study. The events coordinated by SMCs are regulated both by mechanical stretching (111–113) and by the inflammatory cascade that accompanies the process of labor (3, 17, 97, 104, 111, 114–120). The latter concept is supported by in vitro studies showing that CAPs can regulate the expression of inflammatory cytokines in human SMC lines (121). Consistent with these findings, in this study we have shown that the biological processes enriched in SMCs include cytokine signaling pathways containing IL-2, IL-4, TGF-β, and VEGF, as well as IL-6 and IL-1β, classical pathways activated during parturition (122, 123). Indeed, our cell-cell communication analysis indicated that SMCs serve as sensors of such cytokines. Furthermore, we report that specific types of SMCs (e.g., SMC-2 and SMC-3) participate in neutrophil-mediated and IFN-mediated processes, which have been associated with the normal process of labor (11, 124). Yet, to our knowledge, this is the first demonstration that SMCs participate in such processes. Together, these data support a role for uterine SMCs in regulating contractile and inflammatory processes that allow for the physiological transition from a quiescent to an activated state that is required during normal parturition.

In the current study, we found that stromal cells were highly represented among cell types in the myometrial tissues and that such cells displayed a high degree of transcriptomic activity enriched for cell-cell adhesion, leukocyte migration, and wound healing processes. Stromal cells can function as sensors of inflammation (125), promoting both pro- and antiinflammatory processes that depend on the local microenvironment (125, 126). Interestingly, as part of their antiinflammatory properties, mesenchymal stromal cells can produce PGE_2_, which is a strong inducer of myometrial SMC contraction (127–131), in response to inflammatory stimuli such as IFN-γ and TNF (125, 132, 133). This evidence, along with the cell-cell communication results reported herein, in which we showed that stromal cells participate in the contraction pathway, suggest that myometrial stromal cells not only participate in the inflammatory milieu accompanying labor but also support the contraction of SMCs by producing prostaglandins and other prolabor mediators.

Myometrial endothelial cells were another cell type whose transcriptomic activity was highly modulated by the process of labor. Endothelial cells line the internal surface of blood vessels and regulate multiple factors such as cell adhesion, vascular tone, thromboresistance, and vessel wall inflammation that contribute to normal vascular homeostasis (134–136). Moreover, these cells play a central role at sites of acute inflammation (136). It has been suggested that uterine endothelial cells promote the labor-associated infiltration of leukocytes into the uterine tissues (7, 11–15) to propagate inflammation (97). Herein, we put forward evidence for an immune role for 2 types of endothelial cells in the human myometrium: Endothelial-1 participates in antigen presentation, whereas Endothelial-2 is involved in IFN signaling and seems to be more transcriptionally active. Interestingly, cell-cell communication analysis revealed that Endothelial-2 contributes to the pathway of contraction. However, the specific roles of the different clusters of endothelial cells in the myometrium warrant further investigation.

Notably, in the current study, monocytes and macrophages were the primary immune cell subsets modulated by the process of spontaneous labor at term in the myometrial tissues. Macrophages have been demonstrated to be one of the primary inflammatory cell types infiltrating the human myometrium during spontaneous labor at term and are more abundantly found in the lower uterine segment than the upper segment (11). The proinflammatory role of uterine macrophages in spontaneous labor at term is supported by human studies showing that the influx of these innate immune cells coincides with an increased expression of inflammatory mediators such as IL-1β, IL-6, and IL-8 in the human lower uterine segment (14) as well as monocyte chemoattractant protein-1 (MCP-1 or CCL2) in the murine myometrium (16). Furthermore, in vitro evidence suggests that, besides serving as a source of proinflammatory cytokines, monocytes and macrophages can augment myocyte contraction (137). In the current study, we provide further insight into the biology of these innate immune cells. First, we show that both monocytes and macrophages are present in the myometrium and actively participate as both sources and responders of cytokine signaling, the latter revealed by our cell-cell communications analysis. We also report that different myometrial macrophage cell types can be defined according to their transcriptomic signature (Macrophage-1, antigen presentation; Macrophage-2, wound healing; Macrophage-3, neutrophil response; and Macrophage-4, response to bacterium). These data show that the functions of myometrial macrophages are not limited to being sources of cytokines but rather play diverse immune roles in late pregnancy, which may occur in parallel with, or even precede, the process of labor. Indeed, a mechanistic study has shown that maternal macrophages, including those at the materno-fetal interface and myometrium, are required for late pregnancy maintenance (67).

Importantly, we demonstrated herein that single-cell gene signatures derived from the uterine tissues can be evaluated in the maternal circulation, providing a potential biomarker for monitoring changes in the reproductive tissues during pregnancy. This strategy originates from pioneering studies showing that cell-free fetal DNA in the maternal circulation (138) had potential as a screening method for fetal conditions such as trisomy 21 (139, 140) and from a prior investigation integrating scRNA-Seq of the placenta with maternal plasma RNA in women with preeclampsia (58). Similarly, we recently performed scRNA-Seq of the placenta and chorioamniotic membranes and demonstrated that multiple placenta-derived single-cell signatures were modulated with gestational age in the maternal circulation, with specific signatures being altered in women with term or preterm labor (62). This finding is consistent with the results reported herein showing that myometrium-derived single-cell signatures are modulated in the maternal circulation as gestation progresses and during the process of labor. These studies provide what may be the first demonstrations that single-cell signatures derived from the intrauterine tissues have potential predictive value in pregnancy disease as well as for monitoring labor status. Future applications may involve the integration of single-cell signatures with bulk omics data sets, which alone provided demonstrable power for evaluating gestational age–dependent and labor-dependent processes that are modulated in the maternal circulation (28, 69, 91, 93, 141, 142). Specifically, the combination of single-cell and bulk data could allow for noninvasive monitoring of changes in specific cell subsets, such as SMCs, that could be used to observe changes associated with the impending onset of labor.

In summary, herein we have utilized scRNA-Seq to establish a single-cell atlas of the human myometrium as well as to characterize the cell type–specific transcriptomic activity as well as cell-cell communications that are modulated during the physiologic process of spontaneous labor at term. Specifically, we provide evidence that labor affects the majority of cell types found in the myometrium, with the most highly modulated being SMCs, monocytes/macrophages, stromal cells, and endothelial cells, all of which include distinct subsets and can serve as sensors and receivers of labor-related signaling. In addition, we report that such nonimmune and immune cells participate in a plethora of biological pathways associated with the contractile and inflammatory processes of spontaneous labor at term. By integrating our single-cell data with an available bulk transcriptomic data set, we also demonstrated agreement between technologies, with the former displaying greater power to identify labor-specific transcripts. Further deconvolution methods highlighted the contribution of SMCs to the uterine contractility, remodeling, and inflammatory processes associated with labor. Notably, we show that myometrium-derived single-cell signatures can be monitored in the maternal circulation throughout pregnancy and are enriched in women with spontaneous labor at term, providing a potential means of noninvasively monitoring pregnancy and its complications. Together, our findings provide insights into the contributions of specific myometrial cell types to the processes that accompany spontaneous labor at term in the human uterine tissues.

## Methods

More information regarding the study design, laboratory procedures, and data analysis is in the Supplemental Methods.

### Data and materials availability.

The data generated in this study are included in the manuscript and/or in the supplemental materials. Supplemental Tables 9–11 include data pertinent to the scRNA-Seq analysis and are cited in the Supplemental Methods. The scRNA-Seq data reported in this study have been submitted to the NIH dbGAP repository (accession number phs001886.v4.p1). All software and R packages used herein are detailed in Supplemental Methods. Scripts detailing the single-cell analyses are also available at https://github.com/piquelab/ParturitionMyometrium (commit ID 6bc5df23eb8e445ce2487a6e08f4a5c2b4a1e529).

### Study approval.

The collection and use of human materials for research purposes were approved by the Institutional Review Boards of Wayne State University and the NICHD (WSU IRB 031318MP2F). Prior to sample collection, written informed consent was provided by all participating women.

## Author contributions

RPR conceived the study; investigated; performed formal analysis and data curation, validation, and visualization; wrote the original draft; and reviewed and edited the draft. RR conceived the study, investigated, provided resources, reviewed and edited the draft, and acquired funding. VGF investigated, designed methodology, visualized data, and wrote, reviewed, and edited the draft. AP performed formal analysis, performed data curation and visualization, and wrote, reviewed, and edited the draft. ALT performed formal analysis and data curation and validation and reviewed and edited the draft. EP investigated, designed methodology, visualized data, and wrote, reviewed, and edited the draft. JG investigated, performed formal analysis, and wrote, reviewed, and edited the draft. DM investigated and wrote, reviewed, and edited the draft. GB performed formal analysis, data curation, and data validation and reviewed and edited the draft. RP, TK, OH, and CP investigated; designed methodology; and reviewed and edited the draft. KM, JRJ, EJ, CDH, and SMB investigated and reviewed and edited the draft. NGL conceived the study, investigated, performed formal analysis, performed data validation, provided resources, performed data visualization, wrote the original draft, and reviewed and edited the draft.

## Supplementary Material

Supplemental data

Supplemental table 3

Supplemental table 4

Supplemental table 5

Supplemental table 6

Supplemental table 7

Supplemental table 8

Supplemental table 9

Supplemental table 10

Supplemental table 11

## Version 1 03/08/2022

Electronic publication
