# Supplementary material for: A single-cell atlas of the myometrium in human parturition
Source: JCI Insight. 2022 Mar 8;7(5):e153921. doi: 10.1172/jci.insight.153921 (PMC8983148; doi:10.1172/jci.insight.153921)
Supplement: Supplemental data [file jciinsight-7-153921-s253.pdf]

SUPPPLEMENTARY MATERIALS for

A Single-Cell Atlas of the Myometrium in Human Parturition

Supplemental Materials and Methods

Supplemental Figure 1. Pathway analysis of differentially expressed genes among different Smooth Muscle Cell (SMC) types.

Supplemental Figure 2. Pathway analysis of differentially expressed genes between Stromal cell types.

Supplemental Figure 3. Pathway analysis of differentially expressed genes among Macrophage cell types.

Supplemental Figure 4. The effect of labor on a subset of smooth muscle cells in the human myometrium.

Supplemental Figure 5. Top 10 labor-associated differentially expressed genes in selected cell types from the human myometrium.

Supplemental Figure 6. Overlapping cell types of the human myometrium based on labor-associated differentially expressed genes.

Supplemental Figure 7. Pathway analyses of labor-associated genes in all myometrial cell types.

Supplemental Figure 8. Network representation of predicted protein-protein interactions in the human myometrium.

Supplemental Figure 9. Cell type-specific KEGG pathway analysis of labor-associated genes in the human myometrium.

Supplemental Figure 10. Cell type-specific Reactome pathway analysis of labor-associated genes in the human myometrium.

Supplemental Figure 11. Correlation of gene expression changes associated with term labor between the single-cell and bulk transcriptomic data of the human myometrium.

Supplemental Figure 12. Projection of the “SMC-1” signature to infer the underrepresented functions of smooth muscle cell types in labor.

Supplemental Figure 13. Genes and pathways involved in smooth muscle cell physiology of the human myometrium.

Supplemental Table 1. Clinical and demographic characteristics of the study population.

Supplemental Table 2. Summary of cell populations identified in the human myometrium at term pregnancy.

Provided as separate files:

Supplemental Table 3. List of marker genes used for pathway analysis based on Gene Ontology (GO) terms to differentiate Smooth Muscle Cell (SMC) subsets.

Supplemental Table 4. List of marker genes used for pathway analysis based on Gene Ontology (GO) terms to differentiate stromal cell subsets.

Supplemental Table 5. List of marker genes used for pathway analysis based on Gene Ontology (GO) terms to differentiate macrophage subsets.

Supplemental Table 6. List and summary of labor-associated differentially expressed genes (DEGs) in human myometrial cell types.

Supplemental Table 7. Summary of genes and pathways used in the intercellular communication analysis using CellChat.

Supplemental Table 8. Summary of the top 20 genes used to generate signature analysis plots in the comparative analysis between maternal peripheral blood collected throughout gestation and myometrial tissues collected at term.

Supplemental Table 9. Summary of quality control metrics calculated for each library and myometrial sample from the 10x Genomics Cell Ranger pipeline.

Supplemental Table 10. List of marker genes for the identification of cell types in the human myometrium.

Supplemental Table 11. Labor-associated differentially expressed genes in cell types that can be detected in the maternal peripheral blood.

SUPPLEMENTAL MATERIALS AND METHODS

Study design

This prospective cross-sectional study included women that underwent cesarean section at term (≥ 37 weeks of gestation) and were divided into the following groups: term not in labor (N = 13) and term in spontaneous labor (N = 11). The demographic and clinical characteristics of the study groups are shown in Supplemental Table 1. Myometrial biopsies were collected from eligible women at term enrolled in our research protocols at the Detroit Medical Center, Wayne State University School of Medicine, and the Perinatology Research Branch, an intramural program of the *Eunice Kennedy Shriver* National Institute of Child Health and Human Development, National Institutes of Health, U.S. Department of Health and Human Services (NICHD/NIH/DHHS), Detroit, MI, USA.

Laboratory Procedures

Sample Collection

Immediately after the delivery of the placenta, myometrial biopsies were obtained from the lower uterine segment at the time of cesarean section. Biopsies were obtained from the midpoint of the superior edge of the uterine incision and transported to the laboratory in Dulbecco's Modified Eagle Medium (DMEM; Life Technologies Corporation, Grand Island, NY, USA) for single-cell dissociation and formalin fixation/paraffin embedding for further histological characterization. Additionally, a fraction of the myometrial biopsy and samples from the umbilical cord tissues were obtained, snap-frozen in liquid nitrogen, and kept at -80°C for maternal and fetal genotyping, respectively. Placentas were also collected and processed for histological evaluation.

Histological characterization of the myometrial tissues

Hematoxylin & eosin staining

Formalin-fixed paraffin-embedded uterine tissues were cut in 5-µm-thick sections. Slides were deparaffinized in xylene and rehydrated with a series of decreasing ethanol concentrations. Tissue sections were stained with Richard-Allan Scientific Modified Harris Hematoxylin and Eosin Y (both from Thermo Fisher Scientific, Waltham, MA, USA). Brightfield images were taken using the Vectra Polaris Multispectral Imaging System (Akoya Biosciences, Inc., Marlborough, MA, USA).

Smooth muscle actin (SMA) immunohistochemistry

Formalin-fixed paraffin-embedded uterine tissues were cut in 5-µm-thick sections. Slides were deparaffinized in xylene and rehydrated with a series of decreasing ethanol concentrations. Immunohistochemistry staining for smooth muscle actin (SMA, Monoclonal Mouse Anti-Human SMA Clone 1A4; Cat. No. M0851, Dako, Glostrup, Denmark) was performed using the Leica Bond Max automatic staining system (Leica Microsystems, Wetzlar, Germany). The Bond™ Polymer Refine Detection Kit (Leica Microsystems) was used to detect the chromogenic reaction of horseradish peroxidase upon oxidation of 3'3-Diaminobenzidine (DAB) substrate. The mouse negative control antibody (FLEX Universal Negative Control, Agilent, Santa Clara, CA, USA) was used as negative control. Brightfield images were taken using the Vectra Polaris Multispectral Imaging System.

Masson's trichrome staining

After deparaffinization and rehydration as described above, 5-µm-thick myometrial tissue sections were stained using the Masson's 2000 Trichrome Stain Kit (Cat. No. KTMTR2; American MasterTech, Lodi, CA), according to the manufacturer's protocol. Following staining, the sections were dehydrated with increasing concentrations of ethanol and then a coverslip was placed. Brightfield images were taken using the Vectra Polaris Multispectral Imaging System.

Multiplex immunofluorescence

Five-µm-thick sections of formalin-fixed, paraffin embedded myometrial tissues were cut and mounted on microscope slides. Using OPAL Multiplex 7-color IHC kit (Cat. no. NEL811001KT; Akoya Biosciences), myometrial tissue slides were stained according to manufacturer's instructions. Prior to multiplex immunofluorescence staining, each analyte was individually optimized by single-plex antibody staining combined with different fluorescent TSA reagents (Akoya Biosciences). Briefly, after slides were deparaffinized and rehydrated, antigen retrieval (AR) was performed using AR buffer and boiling using a microwave oven. The slides were then incubated in blocking buffer to eliminate non-specific binding prior to incubation with selected primary antibody at room temperature. Next, the slides were rinsed in TBST prior to incubation with anti-mouse and anti-rabbit secondary antibody-HRP conjugate followed by selected fluorescent TSA reagents. Antigen detection steps were performed sequentially using antibodies in the following order: SMA; OXTR (Oxytocin receptor; Polyclonal Rabbit Anti-Human OXTR; Cat. No. LS-A244-50; LifeSpan BioSciences, Seattle, WA, USA); interferon gamma (IFNγ; Polyclonal Rabbit Anti-Human IFNγ; Cat. No. 15365-1-AP, Thermo Fisher Scientific); Neutrophil Elastase (Polyclonal Rabbit Anti-Human Neutrophil Elastase; Cat. No. ab68672; Abcam, Waltham, MA, USA). At the end of the sequential staining of antigens, slides

were incubated with DAPI (4′,6-diamidino-2-phenylindole) as nuclear counterstain and mounted using AquaSlip™ Aqueous Permanent Mounting Medium (American MasterTech). Depending on the host species of the primary antibody, mouse or rabbit isotype (FLEX Universal Negative Control, both from Agilent) was used as negative control. Multiplex staining was performed by consecutively staining slide-mounted tissues using the same antibody concentrations and conditions validated through single-plex staining. Immunofluorescence images were taken using the Vectra Polaris Multispectral Imaging System.

Placental histopathological examination

Placentas were obtained at the time of cesarean section and examined histologically by perinatal pathologists blinded to clinical diagnoses and obstetrical outcomes, according to standardized Perinatology Research Branch protocols (1). Briefly, three to nine sections of the placenta were examined, and at least one full-thickness section was taken from the center of the placenta; others were taken randomly from the placental disc. Acute inflammatory lesions of the placenta were diagnosed according to established criteria (2-4), as shown in Supplemental Table 1.

Myometrial tissue dissociation

Immediately following myometrial biopsy collection, tissues were mechanically and enzymatically homogenized to prepare single-cell suspensions, modified from previously described protocols (5-7). Briefly, myometrial tissues were minced into small pieces until a fine consistency is achieved. Then, the tissues were enzymatically digested using the Umbilical Cord Dissociation Kit (Miltenyi Biotec, San Diego, CA, USA) and incubating at 37°C. The tissues were further dissociated using the gentleMACS Dissociator (Miltenyi Biotec), and the resulting cell suspensions were washed with 1X phosphate-buffered saline (PBS, Thermo Fisher

Scientific/Gibco, Grand Island, NY, USA) and filtered through 100 µm cell strainers (Miltenyi Biotec). Cells were pelleted after centrifugation at 300 x g for 10 min. at room temperature. Erythrocytes were removed using ACK lysing buffer (Life Technologies, Grand Island, NY, USA). Finally, cells were resuspended with 0.04% Bovine Serum Albumin (BSA) (Sigma Aldrich) in 1X PBS and filtered through 30 µm cell strainers (Miltenyi Biotec). Cell concentrations and viability were determined using an automatic cell counter (Cellometer Auto 2000, Nexcelom Bioscience, Lawrence, MA, USA). The Dead Cell Removal Kit (Miltenyi Biotec) was used to remove dead cells to reach a viability of ≥80%.

Cytospin preparations from myometrial cell suspensions

Myometrial tissue biopsies were dissociated as described above. Using a Shandon Cytospin 3 cytocentrifuge (Thermo Fisher Scientific), the resulting single-cell suspensions ($5x10^4$ cells in 200 µL) were cytospun at 800 rpm for 5 min at room temperature onto Fisherbrand Superfrost Plus microscope slide (Thermo Fisher Scientific, Rochester, NY, USA). The slides were fixed with 4% paraformaldehyde (Electron Microscopy Sciences, Hatfield, PA, USA) for 15 minutes and rinsed with 1X PBS prior to H&E and manual SMA immunohistochemistry staining.

Single-cell GEM generation and library construction

Single-cell RNA sequencing library preparation was performed on viable cells using the 10x Genomics Chromium Single Cell 3' Gene Expression Version 3.1 Kit (10x Genomics, Pleasanton, CA, USA), according to the manufacturer's protocol. Briefly, the Chromium Controller was used to generate Gel Bead-in-Emulsions (GEMs) from viable single cell suspensions, whereby a single cell and a single Gel Bead with barcoded oligonucleotides are

encapsulated. Representative images of GEMs were obtained at 200X magnification using an Olympus BX60F microscope (Olympus, Tokyo, Japan) with an Olympus DP71 camera and cellSens Entry version 1.14 software (Olympus). Reverse transcription of mRNA into complementary (c)DNA was performed using the Veriti 96-well Thermal Cycler (Thermo Fisher Scientific, Wilmington, DE, USA), and the resulting cDNA was purified using Dynabeads MyOne SILANE (10x Genomics) and the SPRIselect Reagent (Beckman Coulter, Indianapolis, IN, USA). Enzymatic fragmentation, end-repair, and A-tailing were performed to optimize cDNA amplicon size. Next, adaptors and sample index were incorporated by ligation. The sample index PCR product was then amplified using the Veriti 96-well Thermal Cycler. After performing a post-sample index double-sided size selection using the SPRIselect Reagent kit, the Agilent Bioanalyzer High Sensitivity DNA Chip (Agilent Technologies, Wilmington, DE, USA) was used to analyze and quantify the final library construct as part of quality control.

RNA sequencing

The Kapa DNA Quantification Kit for Illumina platforms (Kapa Biosystems, Wilmington, MA, USA) was used to quantify the post-library constructs prior to sequencing, following the manufacturer’s instructions. The sequencing of 10x scRNA-seq libraries was performed on the Illumina NextSeq 500 at the Genomics Services Center (GSC) of the Center for Molecular Medicine and Genetics (Wayne State University School of Medicine, Detroit, MI, USA). The Illumina 75 Cycle Sequencing Kit (Illumina, San Diego, CA, USA) was used with 58 cycles for R2, 26 for R1, and 8 for I1.

DNA isolation for genotyping

Genomic (g)DNA was extracted from uterine and umbilical cord tissues using the DNeasy Blood and Tissue Kit (Qiagen, Hilden, Germany), following the manufacturer's instructions modified with the addition of 4 µl RNase A (100 mg/mL) (Qiagen) and incubation at 56°C. Purified gDNA samples were quantified using the QubitTM dsDNA HS Assay Kit (Invitrogen, Carlsbad, CA, USA). For genotyping Infinium Global Diversity Array-8 v1.0 Kit microarrays were processed by the Advanced Genomics Core of University of Michigan (Ann Arbor, MI, USA).

Data analysis

1. Genotyping analysis

Genotype information was converted to vcf format using "iaap-cli gencall" and "gtc_to_vcf.py" from Illumina. Imputation to 37.5 M variants using the 1000 Genomes haplotype references was performed using the University of Michigan Imputation Server (https://imputationserver.sph.umich.edu/). The maternal/fetal relationship of the genotyped samples was verified using plink2 KING-robust kinship analysis (8). The vcf files were then filtered for high quality imputation and coverage for at least ten scRNA-seq transcripts using bcftools before they were used for demultiplexing.

2. scRNA-seq data normalization and pre-processing

Sequencing data were processed using Cell Ranger version 4.0.0 (10x Genomics). The fastq files were then aligned using kallisto (9), and bustools (10) was used to summarize the cell/gene transcript counts in a matrix for each sample. In parallel, "cellranger counts" was also used to align the scRNA-seq reads by using the STAR aligner (11) to produce the bam files necessary for demultiplexing the individual of origin based on genotype information using

souporcell (12) and demuxlet (13). The following quality metrics were calculated for each library: the average number of unique molecular identifiers (UMIs), the average number of detected genes, cell count, the average number of reads per cell, the fraction of reads in cells, the percentage of reads mapping to the mitochondrial genome, and valid barcodes across the prepared libraries (Supplemental Table 9). All libraries had excellent quality based on 10X Genomics recommendations. After demultiplexing, we removed any droplet/Gel Bead-in-Emulsion (GEM) barcode that was assigned to doublet or ambiguous cells in demuxlet or souporcell, and only those cells that could be assigned to a pregnancy case were kept. Additionally, we further filtered any cell with less than 100 genes or more than 10,000 genes detected or that had > 25% mitochondrial reads. All count data matrices were then normalized and combined using the Seurat package in R (Seurat version 4.0.1, R version 4.0.3) (14, 15). The total number of cells per donor is provided in Supplemental Table 9. The first 100 principal components were obtained, and the different libraries were integrated and harmonized using the Harmony package in R version 1.0 (16). The top 30 harmony components were then processed to embed and visualize the cells in a two-dimensional map via the Uniform Manifold Approximation and Projection for Dimension Reduction (UMAP) algorithm (17, 18). A resolution of 0.8 was used to cluster the single cells.

3. Cell type annotation

SingleR (19) package in R version 1.4.1 was used to identify cell types based on their similarities to reference datasets (7, 20, 21) with known labels. SingleR annotates single cells from query data by computing the Spearman coefficient between the single-cell gene expression data and samples from the reference dataset. The correlation is measured only based on the variable genes in the reference dataset. The multiple correlation coefficients per cell type are combined

according to the cell type labels of the reference dataset to assign a score per cell type in the single-cell data. In addition to these previously labeled datasets, a publicly available dataset of 2,700 Peripheral Blood Mononuclear Cells (PBMCs) generated by 10x Genomics (22) was used to annotate the immune single cells. An anchor-based supervised mapping workflow was used to integrate reference and query single-cell samples and annotate query cells based on the shared biological states using the FindTransferAnchors and MapQuery functions from Seurat version 4.0.1. More specifically, the query data were first normalized and scaled using the SCTransform function. Then, the anchors between the query and reference data were found by a supervised principal component analysis (sPCA) (23). sPCA identifies the set of principal components that maximizes the dependency between the reference and query data, where the structure of the multimodal dataset is best captured based on the Hilbert-Schmidt Independence Criterion measure. Next, the labels were transferred by a binary classification model built on the reference cell type labels. Finally, the neighbor sets representing the nearest neighbors from the reference to each query cell were identified. The UMAP projection was performed using the neighbor sets and the query data was projected onto the coordinates of the provided reference UMAP. Using different annotations obtained from the supervised reference-mapping workflows (SingleR and Seurat) on four datasets (7, 20-22), the final cell type labels were assigned based on majority vote. We further confirmed their identity and profile by identifying the top differentially expressed genes (DEGs) and performing functional enrichment analysis (see below). If multiple clusters were assigned to the same consensus cell type, we added a sub-index to that cell type for each different original Seurat cluster: Clusters 0 and 9 were annotated as Stromal-1 and Stromal-2; clusters 12, 17, and 20 were annotated as SMC-1, SMC-2 and SMC-3; clusters 1, 2, 14, and 21 were annotated as Macrophage-1 to Macrophage-4, respectively; and clusters 3 and 15 were annotated as

Endothelial-1 and Endothelial-2. All remaining clusters were assigned a unique cell type identifier (Supplemental Table 2).

4. Differential gene expression for cell type analysis

For this analysis, the differential expression of selected marker genes for each cell type/cluster were identified using the Wilcoxon Rank Sum test and the FindAllMarkers function from Seurat. For this analysis, we first compared each cluster to all cell types (Supplemental Table 10). Additionally, we further analyzed clusters of closely related cell types in three major subgroups: i) smooth muscle cells (Supplemental Table 3), ii) stromal cells (Supplemental Table 4), and iii) macrophages (Supplemental Table 5). For this analysis, we compared each sub-cluster to the others in each sub-group to focus on the smaller differences between each sub-type. We further used the top 100 cell markers [ranked based on $\log_2$(Fold change) and requiring $q < 0.1$] assigned to each sub-cluster to analyze the enrichment of pathways in sub-clusters by performing an over-representation analysis based on the Gene Ontology (GO) database (Supplemental Figure 1-3).

5. Differential gene expression in parturition

The identification of labor-associated DEGs between study groups was performed using the DESeq2 R package version 1.32.0 (24). A term for each library was added to the DESeq2 model to correct for technical batch effects (library identifier). For each combination, we only used samples with more than 20 cells; otherwise, it was treated as a non-observed combination. Cell types found in less than 3 samples per study group in each combination were dropped (Supplemental Table 6 shows all of the DEGs).

6. Gene ontology and pathway enrichment analysis of genes affected by parturition

The clusterProfiler in R version 4.0.4 (25) was used to perform Gene Set Enrichment Analysis (GSEA) and Over-Representation Analysis (ORA) based on the Gene Ontology (GO), Kyoto Encyclopedia of Gene and Genomes (KEGG), WikiPathways (26), and Reactome databases. The functions "enrichPathway", "enrichKEGG", and "gseGO" from "clusterProfiler" were used to perform the ORA and GSEA analyses separately for each list of genes obtained as differentially expressed for each cell type. In ORA analyses, the universe of genes for each cell type was the subset that was expressed at a level sufficient to be tested in differential gene expression analysis. When results are combined across cell types, any genes tested (with a calculated p-value) in any of the cell types are used for the universe. Only ORA results that were significant after correction were reported with $q < 0.05$ being considered statistically significant. STRING analysis was performed using the STRINGdb version 2.4.1 and ggplot2 version 3.3.5 R packages together with the STRING database (https://string-db.org) (27).

7. Multivariate Adaptive Shrinkage (MASH) analysis of parturition associated genes.

A meta-analysis of the measured changes between the two study groups across cell types was performed using the mashr R package version 0.2.50 (28). This approach, referred to as MASH analysis, tests and estimates multiple effects (e.g., genes) in many conditions (e.g., cell types) by allowing sparse effects and correlation among non-zero effects in different conditions. This method performs a condition-by-condition analysis to estimate the effect of each gene and its corresponding error in each cell type. Using the estimates from condition-by-condition results, the empirical Bayes procedure learns the sparsity pattern as well as correlation among effects and aggregates the learned patterns to improve the effect estimates and the corresponding measures of

significance across the different cell types. The metaplot function from the rmeta R package version 3.0 was used to visualize the result of meta-analysis based on selected labor-associated DEGs.

8. Cell-cell communication analysis

CellChat (29) was used to infer the cell-cell communications using the single-cell gene expression data and a database of prior knowledge of the interactions between signaling ligands, receptors, and their cofactors. CellChat database integrates the signaling molecule interaction information from KEGG and experimental studies. The curated interactions are categorized into 299 signaling pathways. Herein, the CellChat database was customized by adding the ligand-receptor interaction information for myometrial contraction, which was not included in the original database. CellChat predicts significant communication between cell groups (clusters) by identifying significantly over-expressed ligands and receptors between the cell groups. It calculates the communication probabilities using a mass action-based model based on the average expression values of a ligand and its cognate receptors from two cell groups. The signaling communication probabilities are modeled based on the proportions of cells in each cell group across all cells, as abundant cell groups are more likely to send stronger signals in comparison to rare groups. In the estimated cell-cell communication network with weights as probabilities, the major signaling roles are identified based on centrality metrics from graph theory. CellChat predicts the key sending and receiving signals between specific cell groups. The top 10% of significant cell-cell communications ($p < 0.05$) were selected across different pathways and visualized using the CellChat R package version 1.1.2, ggalluvial R package version 0.12.3, and ggplot2 R package version 3.3.5. The major sending and receiving signaling roles based on

context-specific pathways across different cell groups were identified using a cut-off of 0.7 when visualizing the connection.

9. Comparison to myometrial bulk transcriptomic studies at term pregnancy

Myometrial microarray bulk data comparing women in labor ($N = 19$) and not in labor ($N = 20$) were obtained from a previous study (30), and the $\log_2$(Fold change) from this previous study were compared to those obtained in the current study across different cell types. The comparison was visualized with scatter plots using the ggplot2 R package version 3.3.3 and Spearman correlation analysis. Additionally, the ranked lists of DEGs from a previous myometrial study and DEGs detected in myometrial tissues from the current study were used to identify enriched cell types in the given list based on GSEA performed using clusterProfiler in R version 3.18.1.

10. Deconvolution analysis

Cibersortx (31), a deconvolution analysis method, was used to de-convolute previously collected bulk myometrium transcriptome data (30). Cibersortx employs a linear support vector regression to impute sample-level gene expressions of different cell types from the bulk dataset using the single-cell signature matrix derived from the current study. The single-cell matrix includes the average cell counts across 16 main cell types: Monocyte, stromal (Stromal-1 and Stromal-2), endothelial (Endothelial-1 and Endothelial-2), NK cell, DC, macrophage (Macrophage-1, Macrophage-2, and Macrophage-3), smooth muscle cell (SMC-1, SMC-2, and SMC-3), LED, Decidual, T cell ($CD4^+$ and $CD8^+$), ILC, Myofibroblast, B cell, Unciliated epithelial, Plasmablast, and EVT.

The comparison of log_2(Fold change) values between women at term in labor and not in labor was calculated based on the negative binomial model implemented in the DESeq2 R package version 1.30.1 (24). The *P*-values were adjusted using false discovery rate (32). The DEGs were selected based on an adjusted *P*-value (q) < 0.1. The combined list of DEGs across cell types from the bulk data after deconvolution, and the DEGs from single-cell analysis on the "smooth muscle cell-1" cluster were used to perform ORA using the clusterProfiler in R version 4.0.4 (25).

11. Comparison to maternal peripheral blood bulk transcriptomic longitudinal data

Longitudinal transcriptomic data from maternal peripheral blood collected throughout gestation (N = 49) were taken from a previous study (33) and used to analyze the gestational age-related changes in average expression of cell type-specific gene signatures (cell type markers) at the time of the blood draw (only non-labor samples were available for the longitudinal analysis). The cell type-specific gene signatures were defined using the top 20 DEGs for each cell type (Supplemental Table 8). The expression of cell type-specific gene signatures was averaged based on the gestational age at sampling. The change trend was identified using a linear mixed-effect model fitted based on the quadratic splines. The significant trends were analyzed using linear mixed-effects models for the cell types defined from myometrial scRNA-seq data.

12. Comparison to maternal peripheral blood bulk transcriptomic studies at term pregnancy

Maternal peripheral blood bulk transcriptomic data from women at term in labor (N = 21) and not in labor (N = 28) were obtained from a previous study (34). The ranked list of DEGs in maternal whole blood and DEGs detected in the myometrial tissues from the current study were used to identify enriched cell types in the given list based on GSEA performed using clusterProfiler

in R version 3.18.1. Overlapping DEGs between the single-cell and bulk transcriptomic datasets are shown in Supplemental Table 11.

13. Statistical analysis of the demographic data

Statistical analyses were performed in SPSS v19.0 (IBM, Armonk, NY, USA) or the R package (as described above). Human demographic data were compared using two-tailed Fisher's exact tests for proportions and Mann-Whitney U-tests for non-normally distributed continuous variables.

REFERENCES

1. Romero R, Kim YM, Pacora P, Kim CJ, Benshalom-Tirosh N, Jaiman S, Bhatti G, Kim JS, Qureshi F, Jacques SM, et al. The frequency and type of placental histologic lesions in term pregnancies with normal outcome. *J Perinat Med.* 2018;46(6):613-30.
2. Redline RW. Inflammatory responses in the placenta and umbilical cord. *Semin Fetal Neonatal Med.* 2006;11(5):296-301.
3. Kim CJ, Romero R, Chaemsaithong P, Chaiyasit N, Yoon BH, and Kim YM. Acute chorioamnionitis and funisitis: definition, pathologic features, and clinical significance. *Am J Obstet Gynecol.* 2015;213(4 Suppl):S29-52.
4. Redline RW. Classification of placental lesions. *Am J Obstet Gynecol.* 2015;213(4 Suppl):S21-8.
5. Xu Y, Plazyo O, Romero R, Hassan SS, and Gomez-Lopez N. Isolation of Leukocytes from the Human Maternal-fetal Interface. *J Vis Exp.* 201599):e52863.
6. Tsang JCH, Vong JSL, Ji L, Poon LCY, Jiang P, Lui KO, Ni YB, To KF, Cheng YKY, Chiu RWK, et al. Integrative single-cell and cell-free plasma RNA transcriptomics elucidates placental cellular dynamics. *Proc Natl Acad Sci U S A.* 2017;114(37):E7786-e95.
7. Pique-Regi R, Romero R, Tarca AL, Sendler ED, Xu Y, Garcia-Flores V, Leng Y, Luca F, Hassan SS, and Gomez-Lopez N. Single cell transcriptional signatures of the human placenta in term and preterm parturition. *Elife.* 2019;8(
8. Manichaikul A, Mychaleckyj JC, Rich SS, Daly K, Sale M, and Chen WM. Robust relationship inference in genome-wide association studies. *Bioinformatics.* 2010;26(22):2867-73.
9. Bray NL, Pimentel H, Melsted P, and Pachter L. Near-optimal probabilistic RNA-seq quantification. *Nat Biotechnol.* 2016;34(5):525-7.
10. Melsted P, Booeshaghi AS, Gao F, Beltrame E, Lu L, Hjorleifsson KE, Gehring J, and Pachter L. Modular and efficient pre-processing of single-cell RNA-seq. *bioRxiv.* 2019:673285.
11. Dobin A, Davis CA, Schlesinger F, Drenkow J, Zaleski C, Jha S, Batut P, Chaisson M, and Gingeras TR. STAR: ultrafast universal RNA-seq aligner. *Bioinformatics.* 2013;29(1):15-21.
12. Heaton H, Talman AM, Knights A, Imaz M, Gaffney DJ, Durbin R, Hemberg M, and Lawniczak MKN. Souporcell: robust clustering of single-cell RNA-seq data by genotype without reference genotypes. *Nat Methods.* 2020;17(6):615-20.
13. Kang HM, Subramaniam M, Targ S, Nguyen M, Maliskova L, McCarthy E, Wan E, Wong S, Byrnes L, Lanata CM, et al. Multiplexed droplet single-cell RNA-sequencing using natural genetic variation. *Nat Biotechnol.* 2018;36(1):89-94.
14. Stuart T, Butler A, Hoffman P, Hafemeister C, Papalexi E, Mauck WM, 3rd, Hao Y, Stoeckius M, Smibert P, and Satija R. Comprehensive Integration of Single-Cell Data. *Cell.* 2019;177(7):1888-902 e21.
15. Hafemeister C, and Satija R. Normalization and variance stabilization of single-cell RNA-seq data using regularized negative binomial regression. *Genome Biol.* 2019;20(1):296.
16. Korsunsky I, Millard N, Fan J, Slowikowski K, Zhang F, Wei K, Baglaenko Y, Brenner M, Loh PR, and Raychaudhuri S. Fast, sensitive and accurate integration of single-cell data with Harmony. *Nat Methods.* 2019;16(12):1289-96.

17. McInnes L, Healy J, and Melville J. 2018:arXiv:1802.03426.
18. Becht E, McInnes L, Healy J, Dutertre CA, Kwok IWH, Ng LG, Ginhoux F, and Newell EW. Dimensionality reduction for visualizing single-cell data using UMAP. *Nat Biotechnol.* 2018.
19. Aran D, Looney AP, Liu L, Wu E, Fong V, Hsu A, Chak S, Naikawadi RP, Wolters PJ, Abate AR, et al. Reference-based analysis of lung single-cell sequencing reveals a transitional profibrotic macrophage. *Nat Immunol.* 2019;20(2):163-72.
20. Vento-Tormo R, Efremova M, Botting RA, Turco MY, Vento-Tormo M, Meyer KB, Park JE, Stephenson E, Polanski K, Goncalves A, et al. Single-cell reconstruction of the early maternal-fetal interface in humans. *Nature.* 2018;563(7731):347-53.
21. Wang W, Vilella F, Alama P, Moreno I, Mignardi M, Isakova A, Pan W, Simon C, and Quake SR. Single-cell transcriptomic atlas of the human endometrium during the menstrual cycle. *Nat Med.* 2020;26(10):1644-53.
22. Hao Y, Hao S, Andersen-Nissen E, Mauck WM, 3rd, Zheng S, Butler A, Lee MJ, Wilk AJ, Darby C, Zager M, et al. Integrated analysis of multimodal single-cell data. *Cell.* 2021;184(13):3573-87 e29.
23. Barshan E, Ghodsi A, Azimifar Z, and Zolghadri Jahromi M. Supervised principal component analysis: Visualization, classification and regression on subspaces and submanifolds. *Pattern Recognition.* 2011;44(7):1357-71.
24. Love MI, Huber W, and Anders S. Moderated estimation of fold change and dispersion for RNA-seq data with DESeq2. *Genome Biol.* 2014;15(12):550.
25. Yu G, Wang LG, Han Y, and He QY. clusterProfiler: an R package for comparing biological themes among gene clusters. *OMICS.* 2012;16(5):284-7.
26. Martens M, Ammar A, Riutta A, Waagmeester A, Slenter DN, Hanspers K, R AM, Digles D, Lopes EN, Ehrhart F, et al. WikiPathways: connecting communities. *Nucleic Acids Res.* 2021;49(D1):D613-D21.
27. Szklarczyk D, Morris JH, Cook H, Kuhn M, Wyder S, Simonovic M, Santos A, Doncheva NT, Roth A, Bork P, et al. The STRING database in 2017: quality-controlled protein-protein association networks, made broadly accessible. *Nucleic Acids Res.* 2017;45(D1):D362-D8.
28. Urbut SM, Wang G, Carbonetto P, and Stephens M. Flexible statistical methods for estimating and testing effects in genomic studies with multiple conditions. *Nat Genet.* 2019;51(1):187-95.
29. Jin S, Guerrero-Juarez CF, Zhang L, Chang I, Ramos R, Kuan CH, Myung P, Plikus MV, and Nie Q. Inference and analysis of cell-cell communication using CellChat. *Nat Commun.* 2021;12(1):1088.
30. Mittal P, Romero R, Tarca AL, Gonzalez J, Draghici S, Xu Y, Dong Z, Nhan-Chang CL, Chaiworapongsa T, Lye S, et al. Characterization of the myometrial transcriptome and biological pathways of spontaneous human labor at term. *J Perinat Med.* 2010;38(6):617-43.
31. Newman AM, Liu CL, Green MR, Gentles AJ, Feng W, Xu Y, Hoang CD, Diehn M, and Alizadeh AA. Robust enumeration of cell subsets from tissue expression profiles. *Nat Methods.* 2015;12(5):453-7.
32. Benjamini Y, and Hochberg Y. Controlling the false discovery rate: a practical and powerful approach to multiple testing. *J Roy Statistic Soc Ser B.* 1995;57(1):289-300.

33. Gomez-Lopez N, Romero R, Hassan SS, Bhatti G, Berry SM, Kusanovic JP, Pacora P, and Tarca AL. The Cellular Transcriptome in the Maternal Circulation During Normal Pregnancy: A Longitudinal Study. *Front Immunol.* 2019;10(2863.

34. Gomez-Lopez N, Romero R, Galaz J, Bhatti G, Done B, Miller D, Ghita C, Motomura K, Farias-Jofre M, Jung E, et al. Transcriptome changes in maternal peripheral blood during term parturition mimic perturbations preceding spontaneous Preterm birthdagger. *Biol Reprod.* 2021.

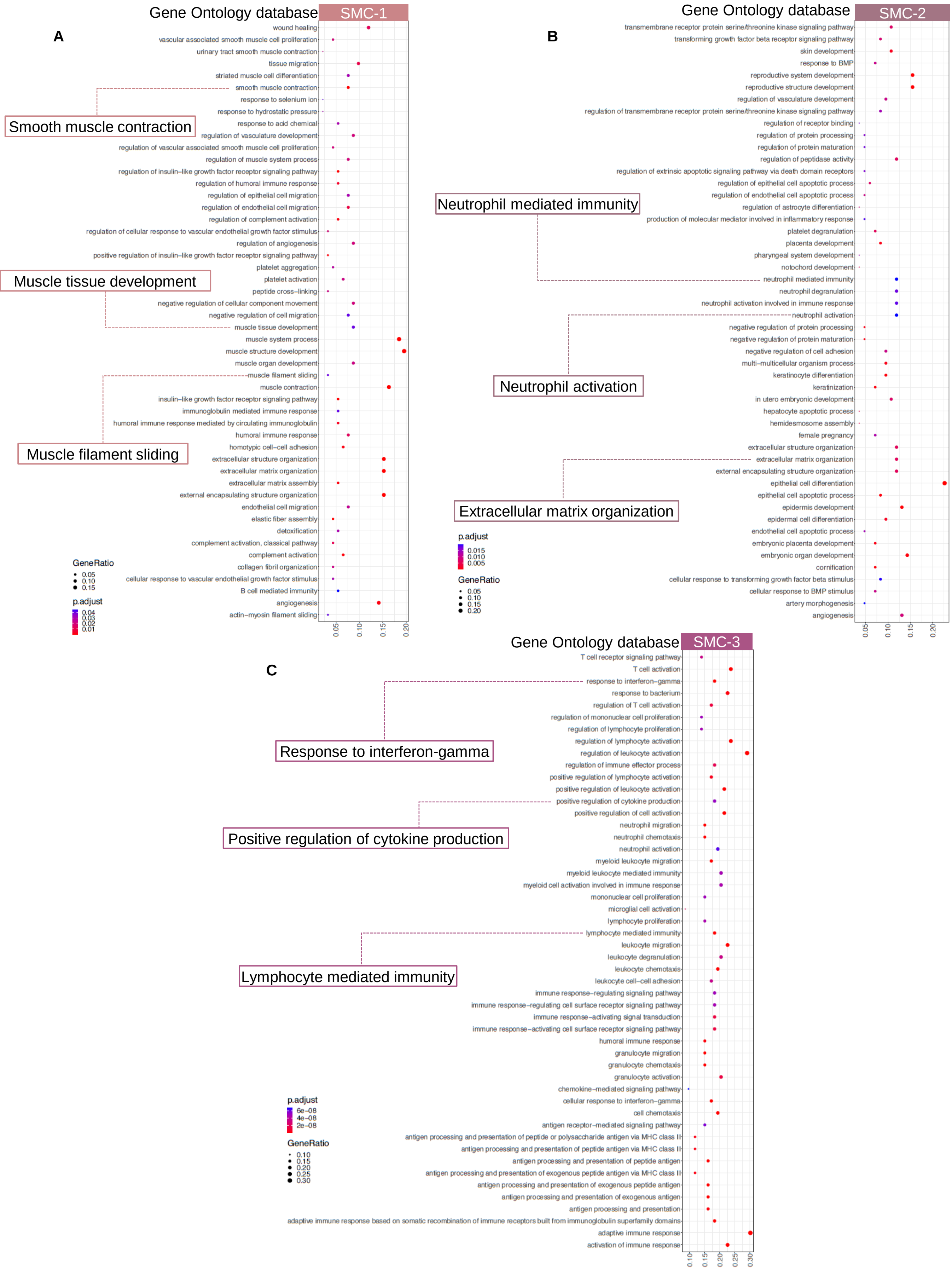

Supplemental Figure 1. Pathway analysis of differentially expressed genes among different Smooth Muscle Cell (SMC) types. ClusterProfiler dot plots showing Gene Ontology (GO) terms enriched for (**A**) SMC-1, (**B**) SMC-2, and (**C**) SMC-3 cell types found in the myometrial tissues (n = 24) based on over-representation analysis (ORA), where the size and color of the dots represent gene ratio and significance level, respectively. Highlighted in cell-type colored insets are pathways that are representative of function in specific SMC subsets. Significant GO terms (q < 0.1) terms were identified based on the one-sided Fisher's exact test.

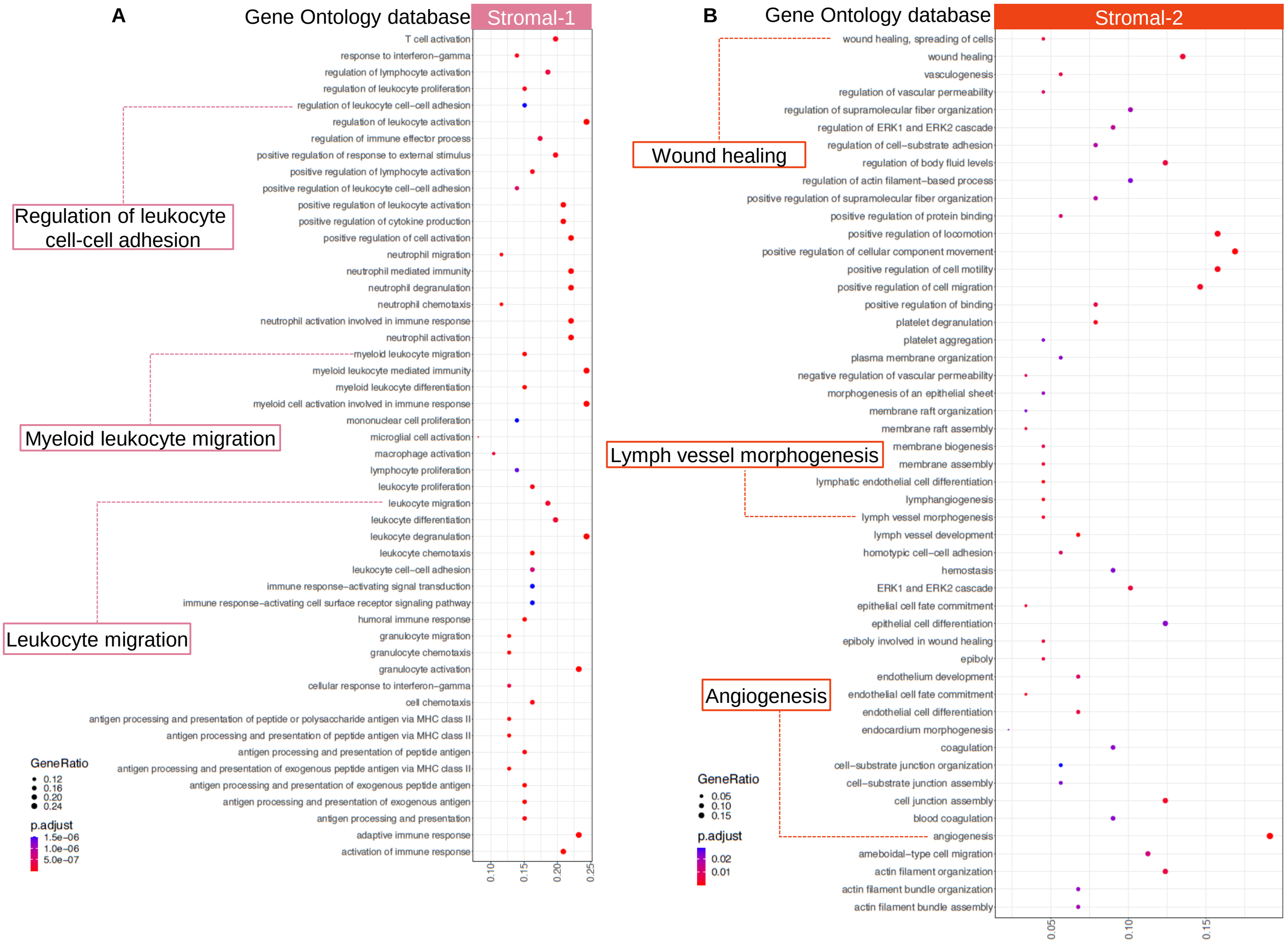

Supplemental Figure 2. Pathway analysis of differentially expressed genes between Stromal cell types. ClusterProfiler dot plots showing Gene Ontology (GO) pathways enriched for (**A**) Stromal-1 and (**B**) Stromal-2 cell types found in the myometrial tissues (*n* = 24) based on over-representation analysis (ORA), where the size and color of the dots represent gene ratio and significance level, respectively. Highlighted in cell-type colored insets are pathways that are representative of function in specific Stromal subsets. Significant GO terms (q < 0.1) were identified based on the one-sided Fisher's exact test.

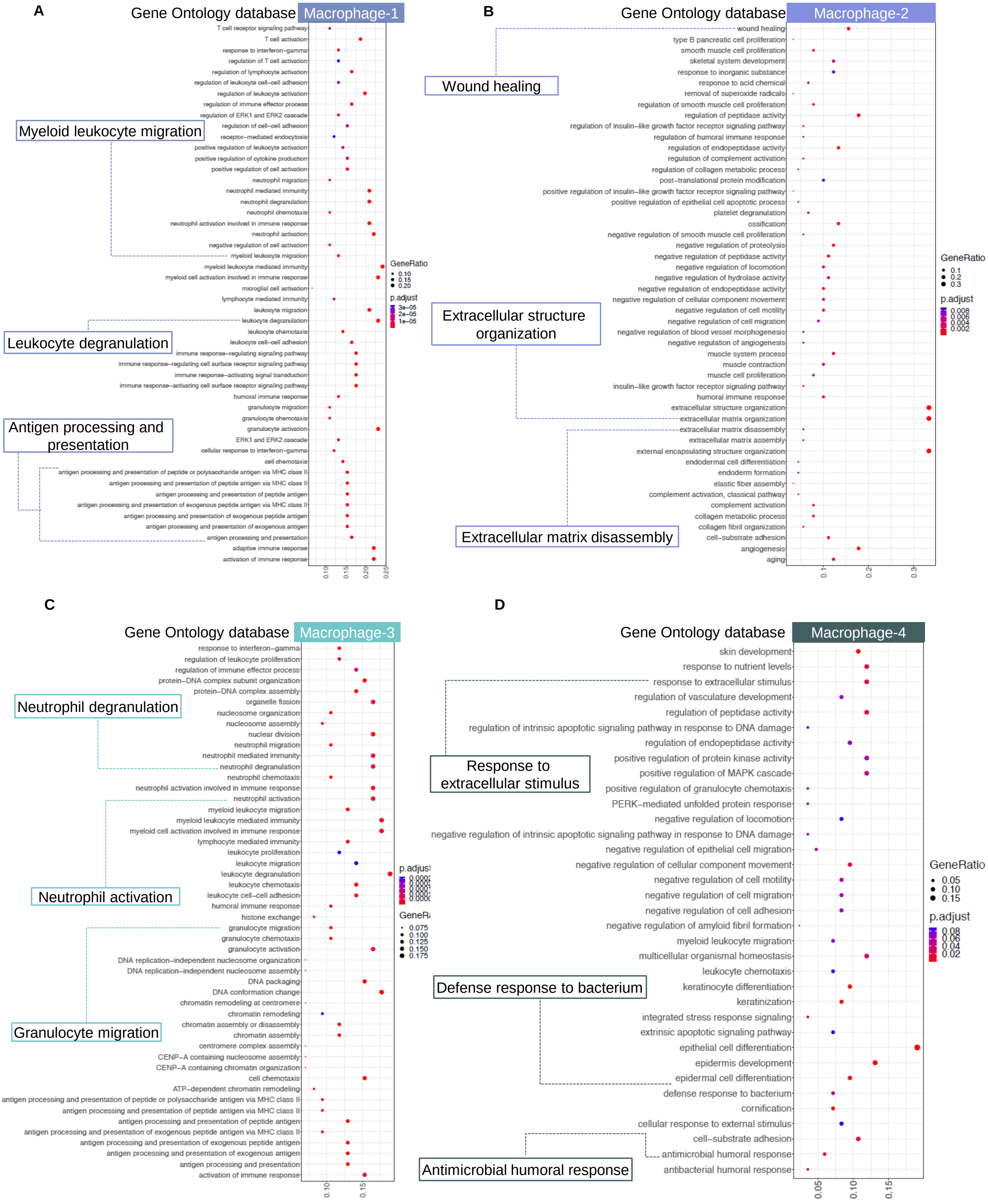

Supplemental Figure 3. Pathway analysis of differentially expressed genes among Macrophage cell types. ClusterProfiler dot plots showing Gene Ontology (GO) pathways enriched for (**A**) Macrophage-1, (**B**) Macrophage-2, (**C**) Macrophage-3, and (**D**) Macrophage-4 cell types found in the myometrial tissues (n = 24) based on over-representation analysis (ORA), where the size and color of the dots represent gene ratio and significance level, respectively. Highlighted in cell-type colored insets are pathways that are representative of function in specific Macrophage subsets. Significant GO pathways (q < 0.1) were identified based on the one-sided Fisher's exact test.

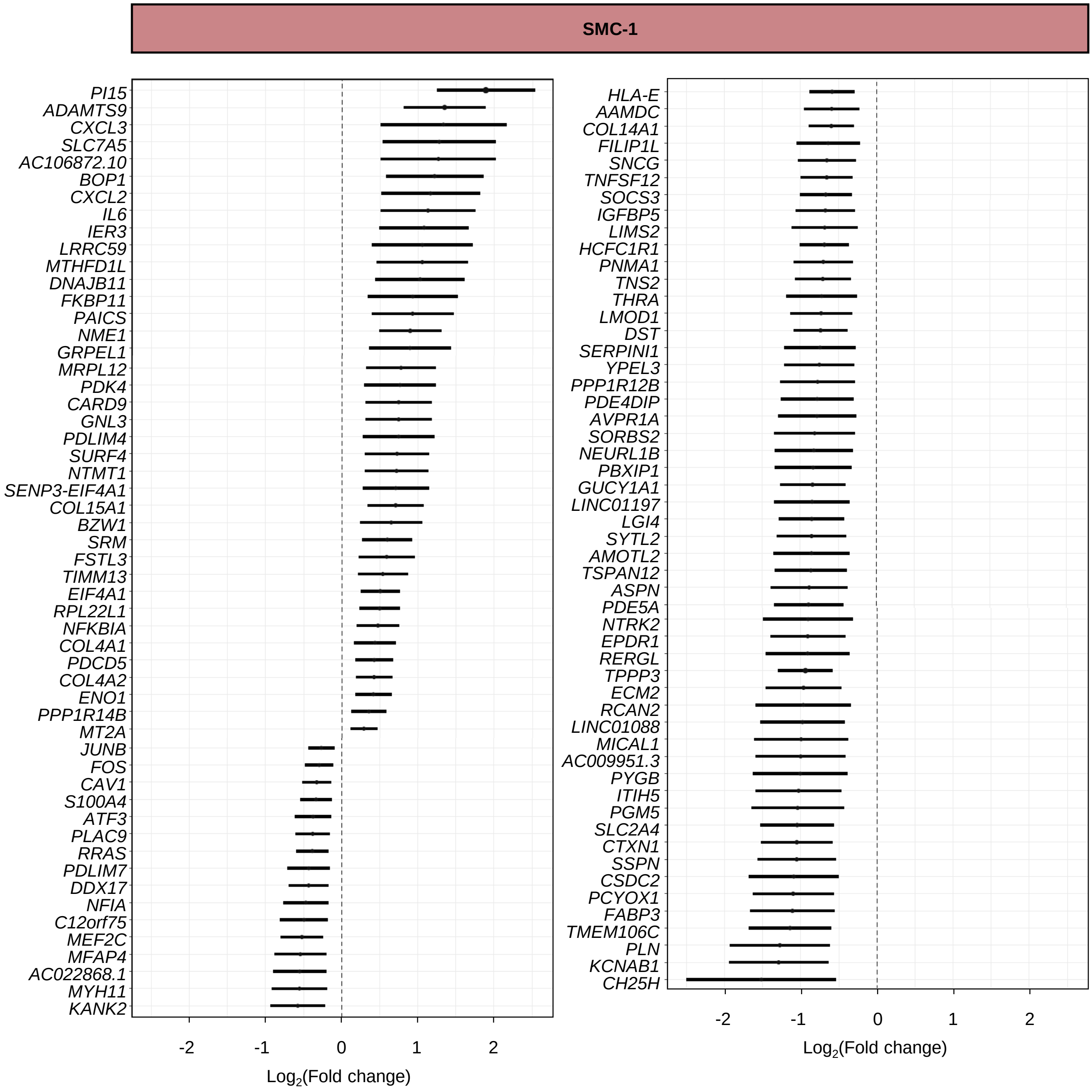

Supplemental Figure 4. The effect of labor on a subset of smooth muscle cells in the human myometrium. Forest plot showing upregulated and downregulated labor-associated differentially expressed genes (DEGs) in Smooth Muscle Cell (SMC)-1 from the myometrial tissues (*n* = 24), where the genes shown are significant with a false discovery rate (FDR) q < 0.05.

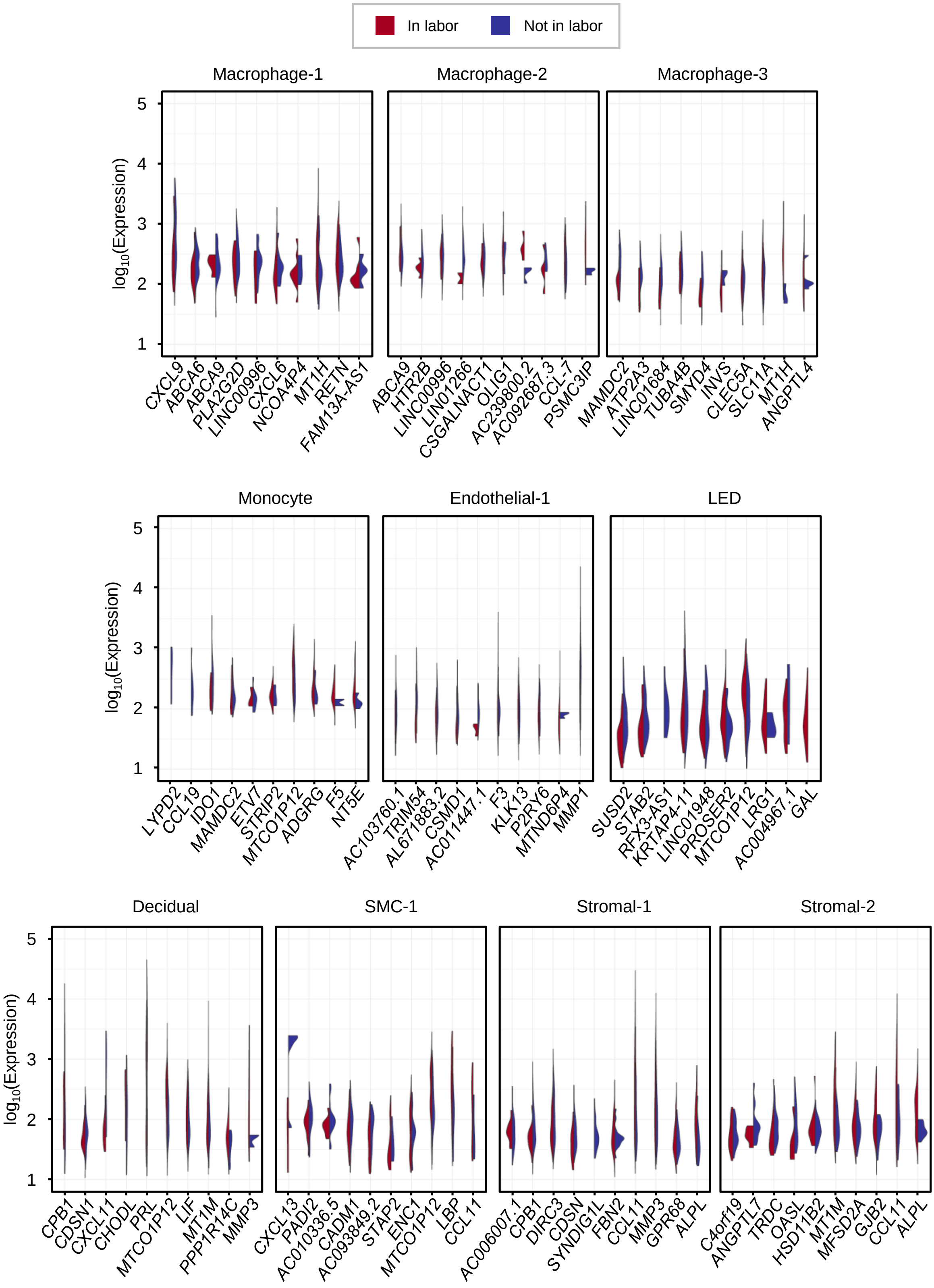

Supplemental Figure 5. Top 10 labor-associated differentially expressed genes in selected cell types from the human myometrium. Violin plots showing the distribution of single-cell gene expression levels for the top ten upregulated and downregulated genes from the myometrial tissues of women in labor (*n* = 11, red) versus not in labor (*n* = 13, blue). Abbreviations used: LED, lymphoid endothelial decidual cell; SMC, smooth muscle cell.

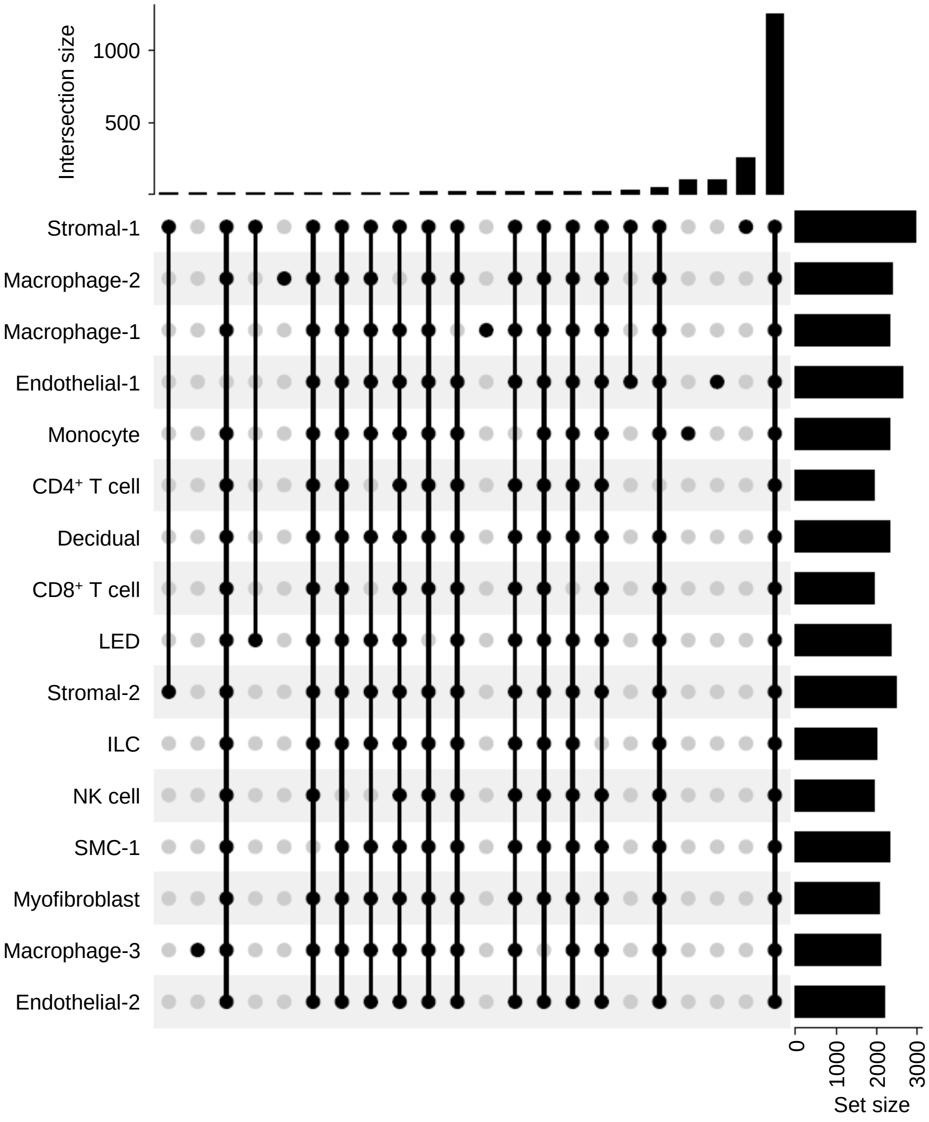

Supplemental Figure 6. Overlapping cell types of the human myometrium based on labor-associated differentially expressed genes. UpSet plot showing intersections between selected cell types in myometrial tissues (*n* = 24) based on the Multivariate Adaptive Shrinkage (MASH) analysis on the $\log_2$(Fold change) of labor-associated differentially expressed genes (DEGs), where connected black dots represent overlapping cell types and vertical bars in intersection set show degree of overlap based on the posterior standard deviation < 0.1. Abbreviations used: ILC, innate lymphoid cell; LED, lymphoid endothelial decidual cell; NK cell, natural killer cell; SMC, smooth muscle cell.

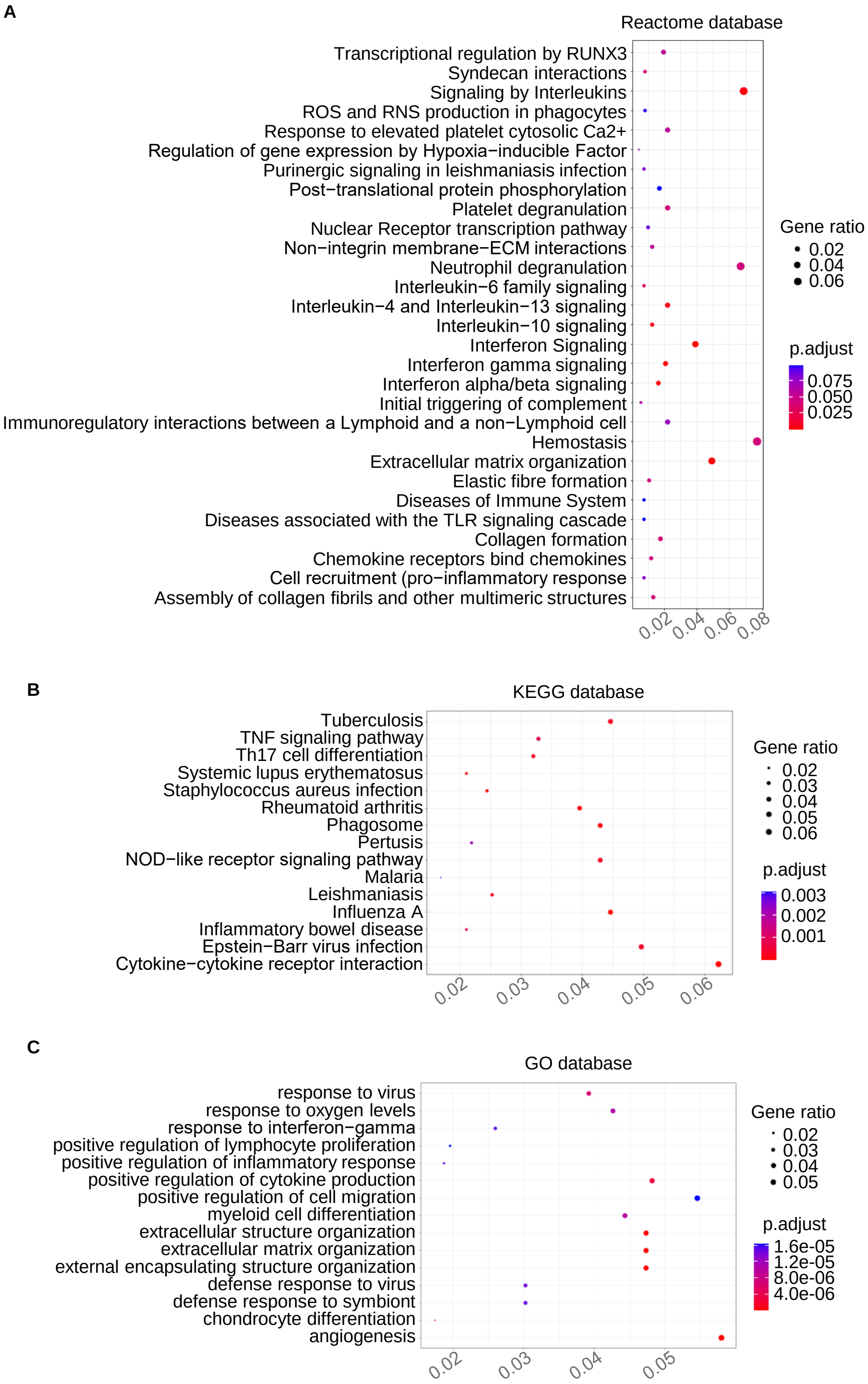

Supplemental Figure 7. Pathway analyses of labor-associated genes in all myometrial cell types. ClusterProfiler dot plots showing (**A**) Reactome pathways, (**B**) Kyoto Encyclopedia of Genes and Genomes (KEGG) pathways, and (**C**) Gene ontology (GO) terms enriched for labor-associated differentially expressed genes (DEGs) in all cell types found in the myometrial tissues (n = 24). Significant pathways (q < 0.05) were identified based on the over-representation analysis using the one-sided Fisher's exact test.

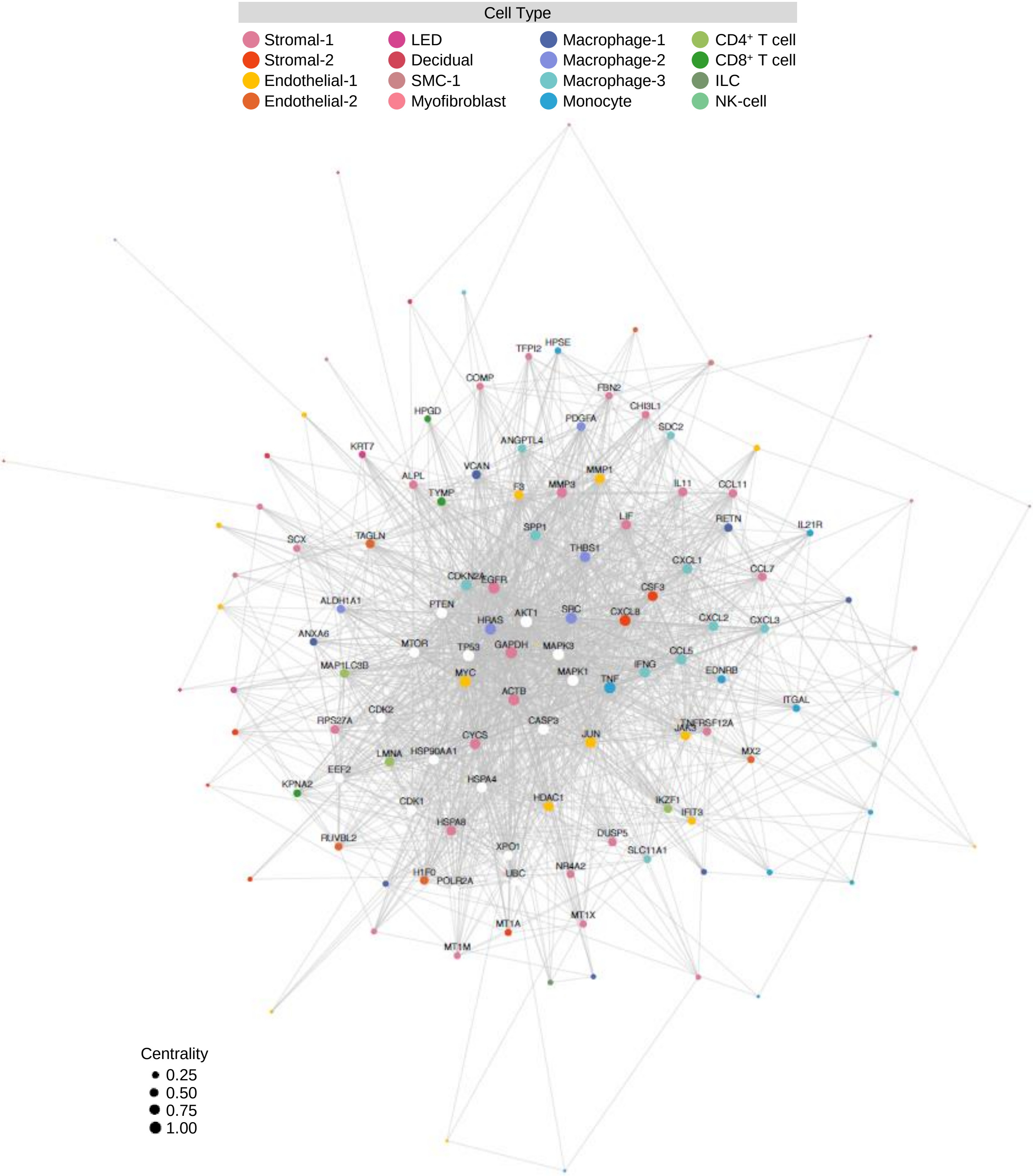

Supplemental Figure 8. Network representation of predicted protein-protein interactions in the human myometrium. Network plot showing predicted protein-protein interactions in the myometrial tissues (n = 24) using the Search Tool for the Retrieval of Interacting Genes/Proteins database (STRINGdb). Each node shows labor-associated differentially expressed genes, where the size is proportional to the degree of centrality and the color represents the myometrial cell type. White-colored nodes represent non-differentially expressed genes with high degree of centrality (hub genes). Abbreviations used: ILC, innate lymphoid cell; LED, lymphoid endothelial decidual cell; NK cell, natural killer cell; SMC, smooth muscle cell.

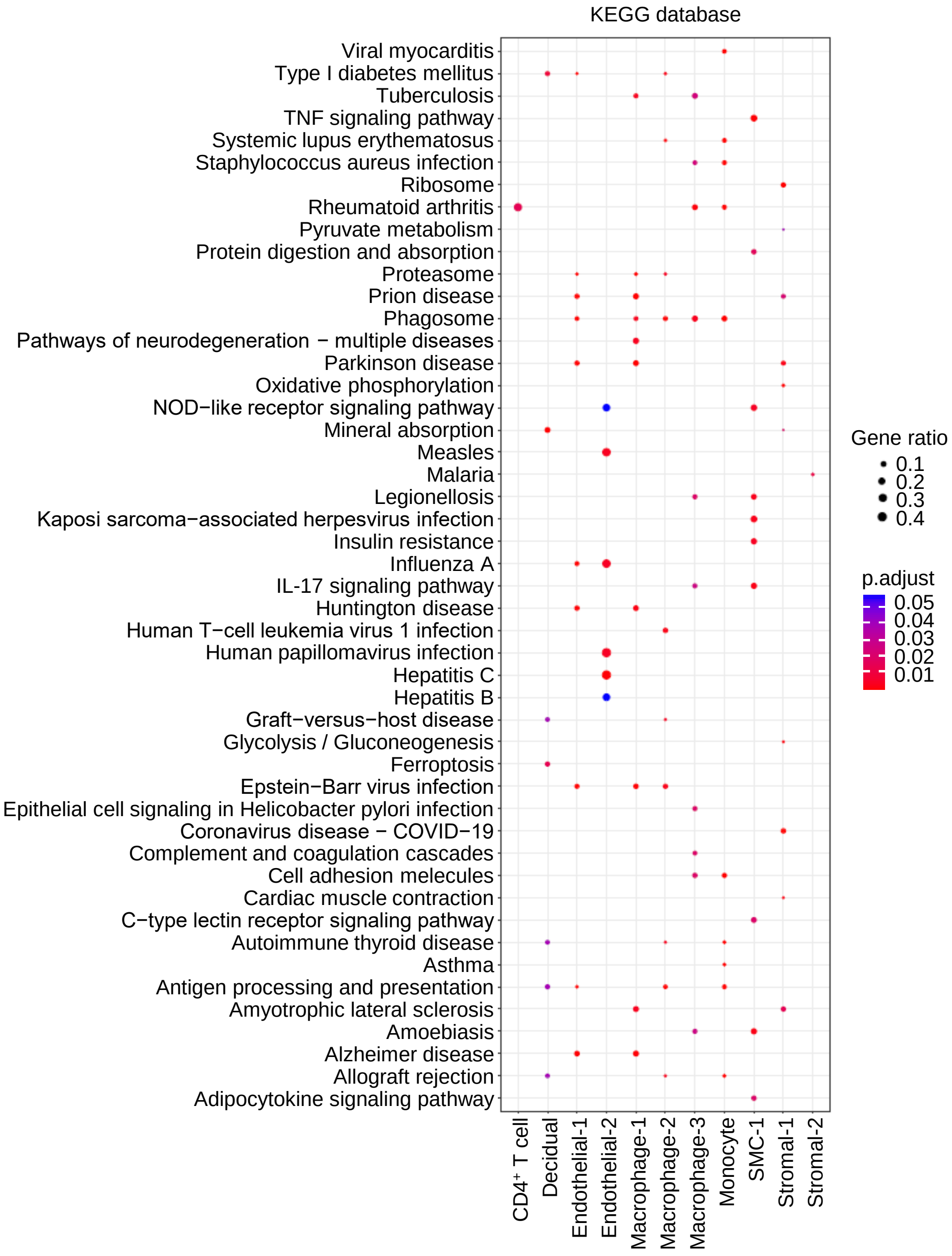

Supplemental Figure 9. Cell type-specific KEGG pathway analysis of labor-associated genes in the human myometrium. ClusterProfiler dot plot showing Kyoto Encyclopedia of Genes and Genomes (KEGG) pathways enriched for labor-associated DEGs in CD4⁺ T cell, Decidual, Endothelial-1, Endothelial-2, Macrophage-1, Macrophage-2, Macrophage-3, Monocyte, Smooth Muscle Cell (SMC)-1, Stromal-1, and Stromal-2 cell types of myometrial tissues (*n* = 24) based on the over-representation analysis, where the size and color of the dots represent enrichment score and significance level, respectively. Significant KEGG pathways (q < 0.05) were identified based on the over-representation analysis using the one-sided Fisher's exact test.

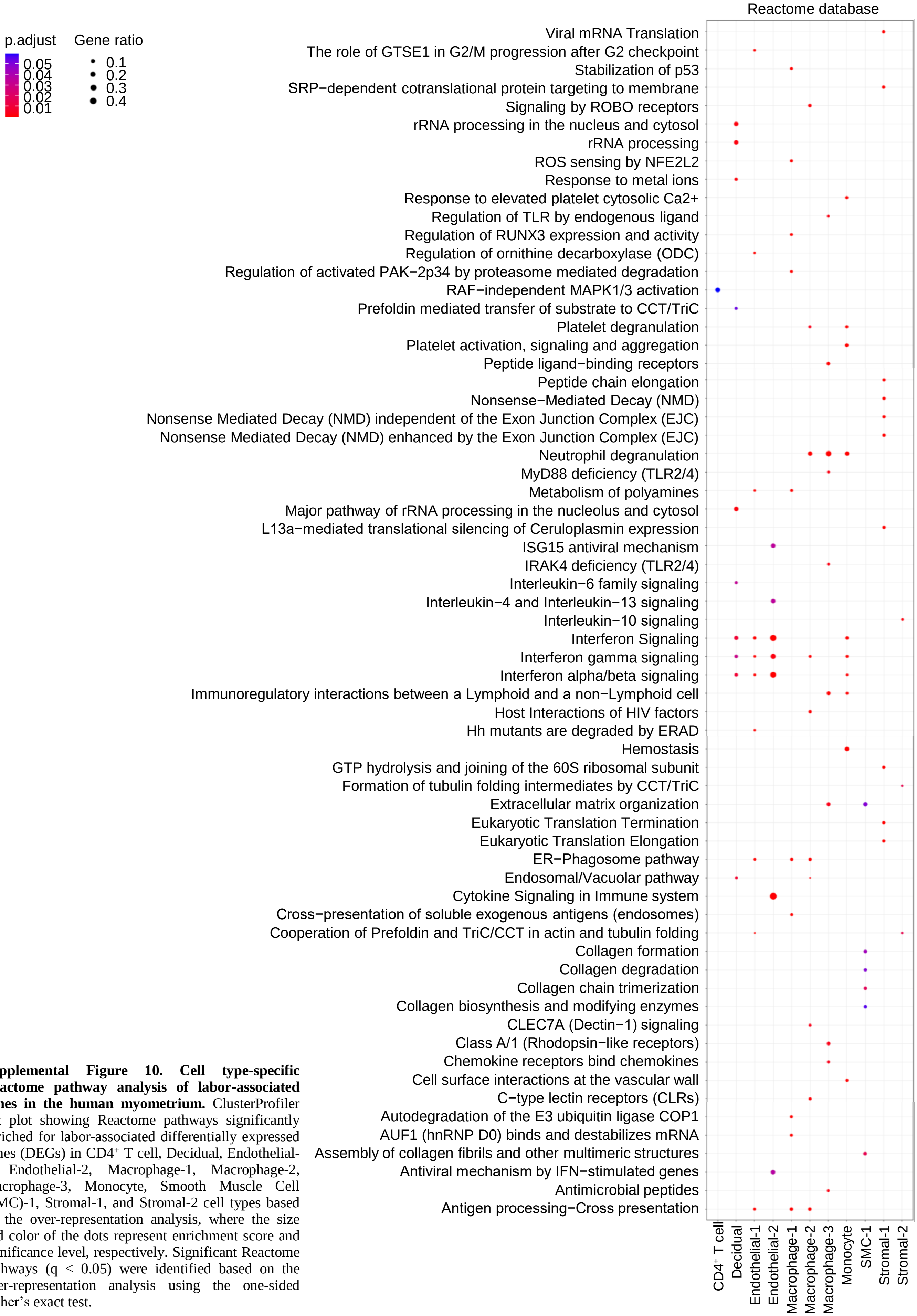

Supplemental Figure 10. Cell type-specific Reactome pathway analysis of labor-associated genes in the human myometrium. ClusterProfiler dot plot showing Reactome pathways significantly enriched for labor-associated differentially expressed genes (DEGs) in $CD4^+$ T cell, Decidual, Endothelial-1, Endothelial-2, Macrophage-1, Macrophage-2, Macrophage-3, Monocyte, Smooth Muscle Cell (SMC)-1, Stromal-1, and Stromal-2 cell types based on the over-representation analysis, where the size and color of the dots represent enrichment score and significance level, respectively. Significant Reactome pathways ($q < 0.05$) were identified based on the over-representation analysis using the one-sided Fisher's exact test.

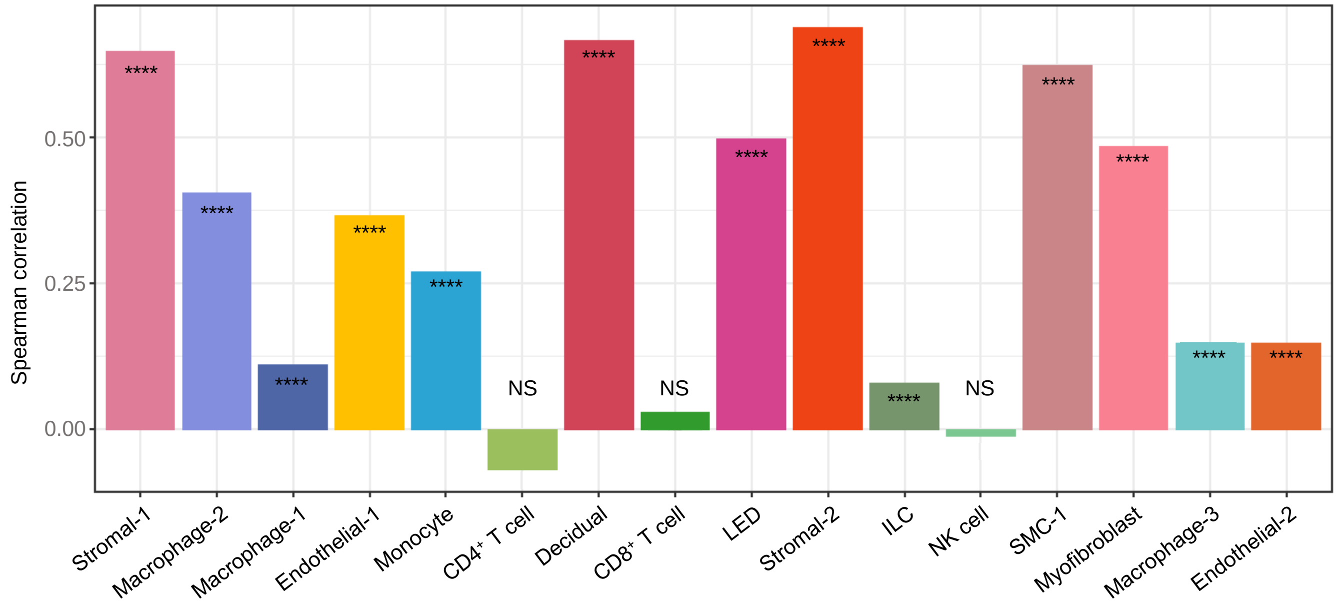

Supplemental Figure 11. Correlation of gene expression changes associated with term labor between the single-cell and bulk transcriptomic data of the human myometrium. Bar plot showing correlations between Log_2(Fold change) of labor-associated differentially expressed genes (DEGs) from single-cell (n = 24) and bulk transcriptomic analyses (n = 39) (significant with $q < 0.1$) using the Spearman correlation test. P-values are considered significant when $P < 0.05$. NS = not significant, * $P \leq 0.05$, ** $P \leq 0.01$, *** $P \leq 0.001$, **** $P \leq 0.0001$. Abbreviations used: ILC, innate lymphoid cell; LED, lymphoid endothelial decidual cell; NK cell, natural killer cell; SMC, smooth muscle cell.

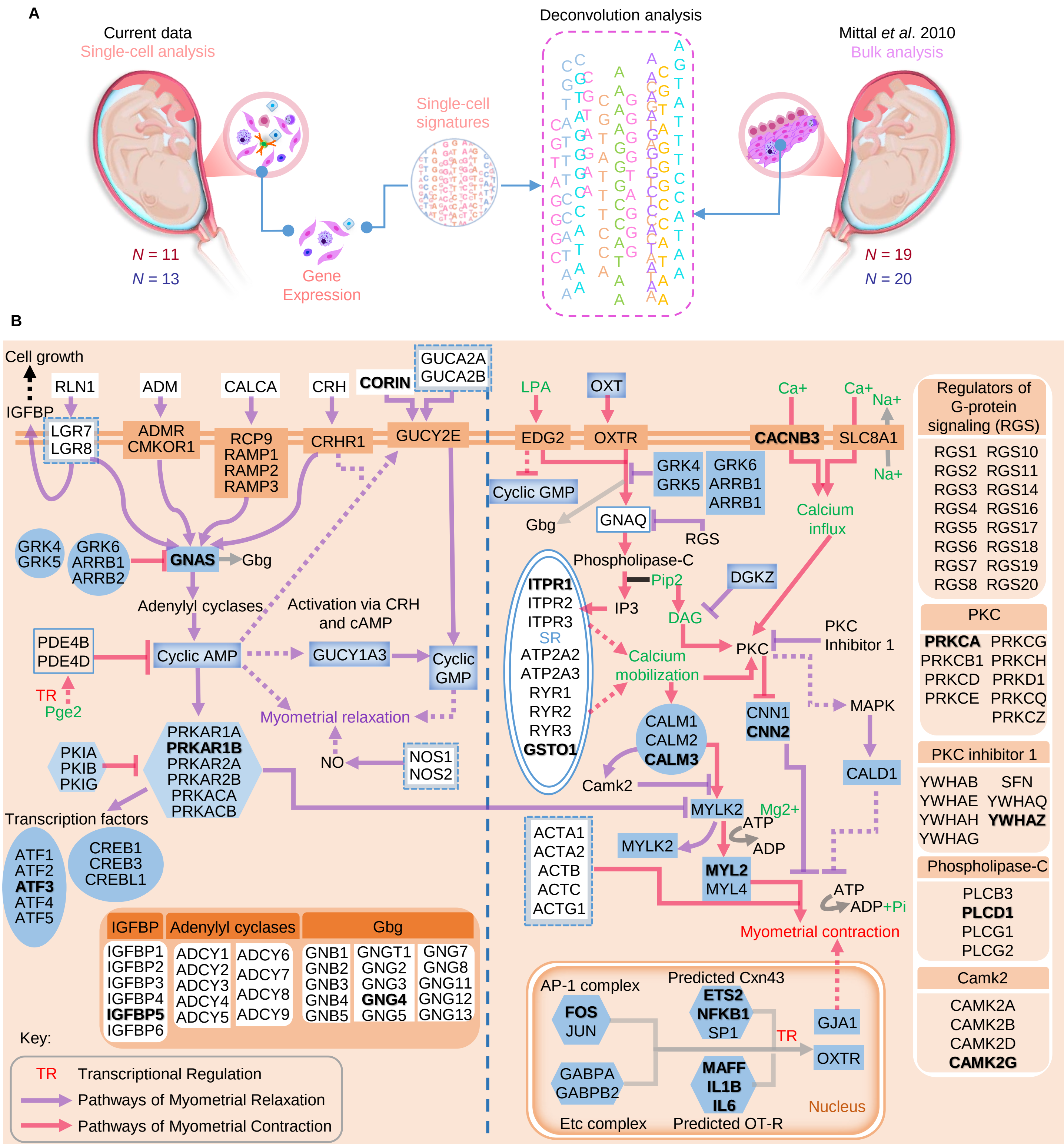

Supplemental Figure 12. Projection of the "SMC-1" signature to infer the underrepresented functions of smooth muscle cell types in labor. (**A**) Diagram illustrating the deconvolution analysis using signatures from the single-cell dataset of the human myometrium in labor (*n* = 11, red) and not in labor (*n* = 13, blue) to obtain differentially expressed genes (DEGs) from inferred cell types in the bulk dataset from myometrial tissues in labor (*n* = 19, red) and not in labor (*n* = 20, blue). (**B**) Visualization of "Myometrial relaxation and contraction pathways" from WikiPathway (https://www.wikipathways.org/index.php/Pathway:WP289) that was significant based on the over-representation analysis of DEGs obtained from deconvolution analysis and single-cell SMC-1 (shown in Fig. 8E). Purple and pink lines depict pathways associated with myometrial relaxation and contraction, respectively. DEGs in bold indicate overlap between WP289 pathway and single cell SMC-1 (shown in Supplementary Figure 13A).

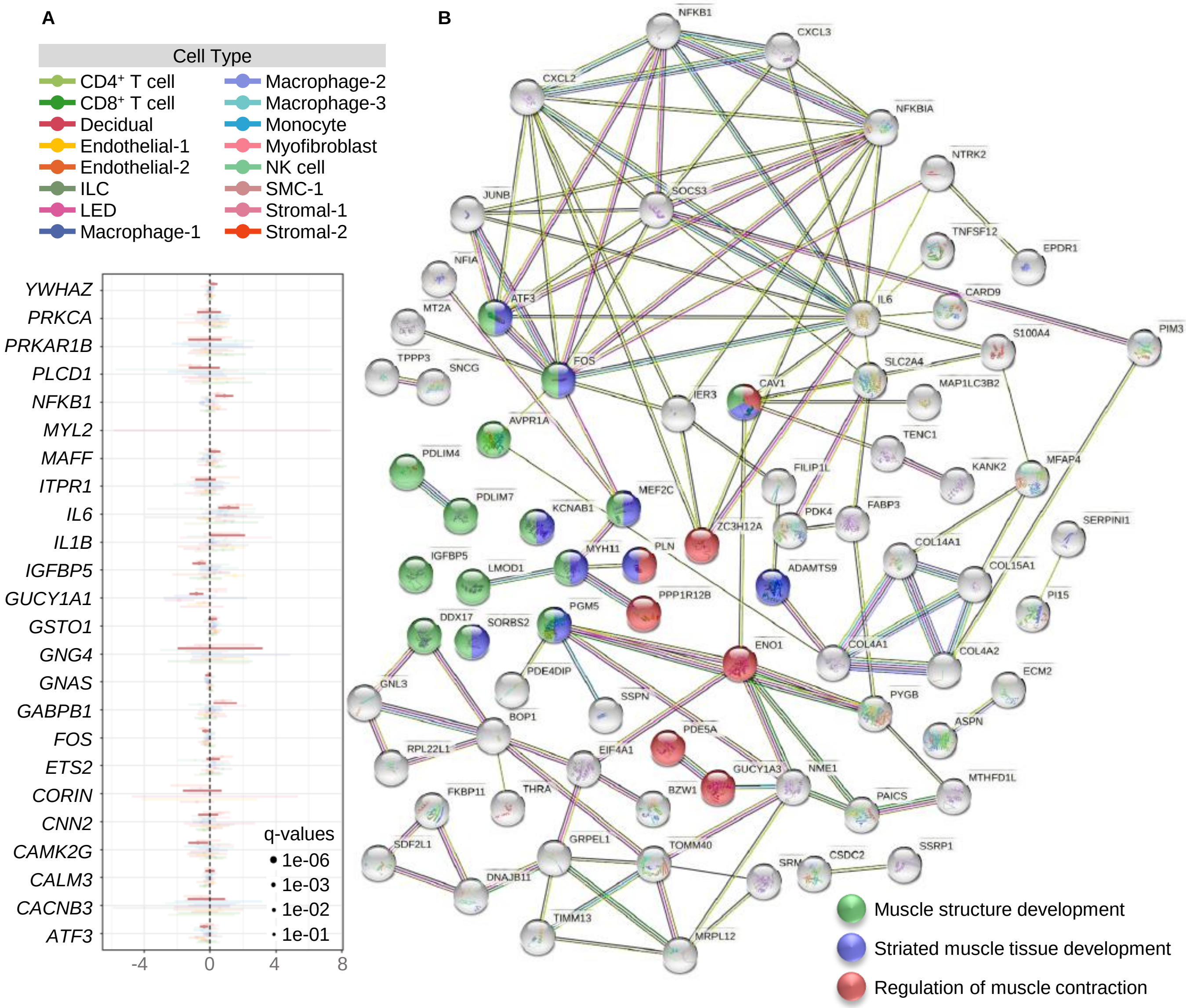

Supplemental Figure 13. Genes and pathways involved in smooth muscle cell physiology of the human myometrium. (**A**) Forest plot showing upregulated and downregulated labor-associated differentially expressed genes (DEGs) from myometrial tissues in labor (n = 11) and not in labor (n = 13) overlapping with the "Myometrial relaxation and contraction pathways" (WP289) q < 0.1. Opaque light pink lines highlight the expression of DEGs in inferred "smooth muscle cell-1", while transparent lines which are color-coded according to cell type represent expression of DEGs in other cell types. (**B**) Search Tool for the Retrieval of Interacting Genes/Proteins (STRING) analysis of labor-associated DEGs (q < 0.1) in "SMC-1" showing the gene interactions of enriched GO terms: muscle structure development (green), striated muscle tissue development (blue), and regulation of muscle contraction (red).

Supplemental Table 1. Clinical and demographic characteristics of the study population. Differences between groups were evaluated by two-sided Mann-Whitney U-test and two-sided Fisher's exact test. P-values < 0.05 were used to denote a significant result.

	**Women without labor at term (*n* = 13)**	**Women with labor at term (*n* = 11)**	***P*-value**
Maternal age (years; median [IQR])[a]	32 (28-36)	29 (25.5-31.5)	0.2
Body mass index (kg/m^2; median [IQR])[a]	32.2 (31.2-36.4)	33.4 (29.4-49.9)	0.6
Primiparity[b]	0% (0/13)	18.2% (2/11)	0.2
Race/ethnicity[b]			1.0
African American	76.9% (10/13)	81.8% (9/11)	
White	7.7% (1/13)	9.1% (1/11)	
Asian	7.7% (1/13)	0% (0/11)	
Not provided	7.7% (1/13)	9.1% (1/11)	
Gestational age at delivery (weeks; median [IQR])[a]	39 (39-39.1)	39.4 (38.5-39.6)	0.4
Birthweight (grams; median [IQR])[a]	3380 (3015-3850)	3360 (2998-3508)	0.6
Apgar score at 1 min (median [IQR])[a]	8 (8-8)	8 (8-8)	0.8
Apgar score at 5 min (median [IQR])[a]	9 (9-9)	9 (9-9)	1.0
Acute maternal inflammatory response[b]			
Stage 1 (Early acute subchorionitis or chorionitis)	7.7% (1/13)	27.3% (3/11)	0.3
Stage 2 (Acute chorioamnionitis)	7.7% (1/13)	18.2% (2/11)	0.6
Stage 3 (Necrotizing chorioamnionitis)	0% (0/13)	0% (0/11)	1.0
Acute fetal inflammatory response[b]			
Stage 1 (Chorionic vasculitis or umbilical phlebitis)	23.1% (3/13)	9.1% (1/11)	0.6
Stage 2 (Umbilical arteritis)	0% (0/13)	9.1% (1/11)	0.5
Stage 3 (Necrotizing funisitis)	0% (0/13)	0% (0/11)	1.0

Data are given as median (interquartile range, IQR) and percentage (n/N).
[a]Mann-Whitney U-test, [b]Fisher's exact test.

Supplemental Table 2. Summary of cell populations identified in the human myometrium at term pregnancy. Table showing different cell types found in the myometrium with corresponding cluster number (as shown in Fig. 1B) and number of cells encapsulated within the 10x Genomics Gel-bead-in-emulsion (GEM).

Cluster number	Cell type	Cell count
**0**	Stromal-1	6,697 cells
**1**	Macrophage-2	4,968 cells
**2**	Macrophage-1	4,205 cells
**3**	Endothelial-1	3,934 cells
**4**	Monocyte	3,528 cells
**5**	$CD4^+$ T cell	3,434 cells
**6**	Decidual	3,311 cells
**7**	$CD8^+$ T cell	3,182 cells
**8**	Lymphoid Endothelial Decidual (LED)	1,914 cells
**9**	Stromal-2	1,709 cells
**10**	Innate Lymphoid Cell (ILC)	1,418 cells
**11**	Natural Killer (NK) cell	1,363 cells
**12**	Smooth Muscle Cell-1 (SMC-1)	1,347 cells
**13**	Myofibroblast	984 cells
**14**	Macrophage-3	530 cells
**15**	Endothelial-2	509 cells
**16**	Dendritic Cell (DC)	440 cells
**17**	Smooth Muscle Cell-2 (SMC-2)	270 cells
**18**	Extravillous Trophoblast (EVT)	259 cells
**19**	Plasmablast	237 cells
**20**	Smooth Muscle Cell-3 (SMC-3)	180 cells
**21**	Macrophage-4	164 cells
**22**	B cell	149 cells
**23**	Unciliated epithelial	112 cells
